# Application of multispectral optoacoustic tomography for lower limb musculoskeletal sports injuries in adults

**DOI:** 10.1016/j.pacs.2024.100656

**Published:** 2024-10-09

**Authors:** Rene B. Svensson, Anne-Sofie Agergaard, Thomas Sardella, Charlène Reichl, Mikkel H. Hjortshoej, Monika L. Bayer, Rikke Hoeffner, Christian Couppé, Michael Kjaer, S. Peter Magnusson

**Affiliations:** a Institute of Sports Medicine Copenhagen, Department of Orthopedic Surgery, Copenhagen University Hospital - Bispebjerg and Frederiksberg, Copenhagen, Denmark; b Center for Healthy Aging, Department of Clinical Medicine, Faculty of Health and Medical Sciences, University of Copenhagen, Copenhagen, Denmark; c Department of Physical and Occupational Therapy, Copenhagen University Hospital - Bispebjerg and Frederiksberg, Copenhagen, Denmark; d iThera Medical GmbH, Munich, Germany; e Centre for Health and Rehabilitation, University College Absalon, Slagelse, Denmark

**Keywords:** Achilles rupture, hemoglobin, lipid, muscle strain injury, patellar tendon, photoacoustic, tendinopathy

## Abstract

Compositional changes in relation to musculoskeletal injuries are difficult to measure non-invasively. This study aims to use non-invasive label-free imaging with Multispectral Optoacoustic Tomography (MSOT) to evaluate compositional changes with injury. Five different patient groups were examined, covering diagnoses of Achilles or patellar tendinopathy, Achilles tendon rupture and gastrocnemius muscle strain injury. Injured and contralateral limbs were imaged using a commercial MSOT device. Hemoglobin, collagen, and lipid contents were estimated. Some patients were examined before and after exercise. Hemoglobin measures had high reproducibility and displayed systematic changes in response to exercise. The content and exercise response of hemoglobin was equal on both limbs. In contrast, collagen and lipid measures were inconsistent and did not display the expected distribution. In conclusion, MSOT is applicable to imaging of hemoglobin in musculoskeletal injuries, providing complimentary information to conventional ultrasound, but applicability to other components like collagen and lipids could not be shown.

## 1 Introduction

Musculoskeletal injuries are common, especially in sports active populations [1], [2], [3], [4]. While the defining features of an injury are the clinical symptoms of pain and dysfunction, most injuries also present with structural or compositional changes of the injured tissue [5], [6], [7], [8], [9]. These changes may provide valuable information on the development, progression and prognosis of the injury.

Tendinopathy is a frequent chronic overuse injury resulting in tendon pain that can persist for years [10]. Structural changes, including ingrowth of blood vessels, often persist after symptom cessation [5], [11], [12], [13] and tendinopathy has a high recurrence rate, suggesting that residual structural changes may predispose to future reinjury.

Achilles tendon rupture is a debilitating injury that recovers slowly and incompletely [14]. During healing the tendon elongates, which likely contributes to loss of function [15], but there is large variation between patients and a less dense collagen matrix has been associated with worse function [16]. The compositional changes responsible for reduced matrix density remain unknown but could include fatty degeneration.

Muscle strain injuries usually occur at the interface of muscles and their connective tissue aponeuroses [8], [17]. While functional recovery is good, permanent structural and compositional changes remain, including swelling of the aponeurosis and fat infiltration of the muscle, which likely contribute to high reinjury rates in these patients [3], [8].

Musculoskeletal tissue changes can be assessed by a multitude of modalities including ultrasound [5], [18], [19], [20], near-infrared spectroscopy (NIRS) [21], laser Doppler flowmetry [22], MRI [7], [23] and biopsies [24].

These methods have some inherent limitations: Non-imaging methods like NIRS may receive signal from surrounding tissues other than the target. Biopsies are invasive and only target a very small region of the tissue. Contrast based methods are invasive and often time restrained due to washout of contrast and limits on the number of injections that can be administered. MRI is time consuming, expensive and may have limited resolution (>1 mm) depending on the sequence. Ultrasound Doppler has limited sensitivity to slow flow and evaluation of B-mode structure is usually not quantitative. Consequently, there is a need for more accurate, valid, and non-invasive methodology for diagnostics of sports-injuries in the musculoskeletal system.

Multispectral Optoacoustic Tomography (MSOT) is a relatively new modality in clinical use with promising features regarding non-invasive assessment of tissue composition [25], [26], [27]. The method uses absorption of optical light in the near-infrared range to generate soundwaves via the optoacoustic effect, which are then received by an ultrasound probe to generate an image of the absorbance of the tissue at a given wavelength. By utilizing multiple different optical wavelengths, an absorption spectrum can be generated, and based on the known spectra of target molecules, it is possible to locate and potentially quantify these. The method can be used in conjunction with contrast agents that have an easily distinguishable absorption spectrum [28], [29] and can be bound to targets of interest. Alternatively, the inherent absorption of tissue constituents may be used to locate and potentially quantify the composition of the tissue in terms of these constituents [30], [31].

The method has been applied in animal studies to investigate a wide range of conditions, including cancer [32] inflammation [33], [34] and metabolic disorders [35], amongst others. Contrast agents are often used, which target specific molecules, but intrinsic signals, e.g. hemoglobin in the vasculature, is also widely used.

More recently, studies using the method in humans have also been reported across several different diagnoses [36], [37], [38]. In human studies, the intrinsic tissue signal is most commonly used due to the strict requirements for contrast injection. Some of these studies have investigated musculoskeletal tissues [31], [39], [40], [41], [42], [43], including fibrosis and vascularity in pediatric muscular dystrophy [31], [39], muscle perfusion dynamics in healthy adults [40], joint and enthesis inflammation with arthritis [41], [42], visualizing different tendons and surrounding vasculature of the hands and feet in 3D and measuring changes in blood oxygenation of the Achilles tendon after physical exercise [43]. All of these outcomes are also highly relevant in overuse and traumatic sports injuries but have not previously been investigated.

The present pilot study aimed to determine the applicability and reproducibility of MSOT for assessing injuries to tendons and muscles in adults, including chronic and recovered tendinopathy of the patellar tendon, early tendinopathy of the Achilles tendon, complete Achilles tendon rupture and calf muscle strain injuries. We also investigated the acute effect of different exercises on MSOT parameters. The molecular targets of interest were: Oxygenated and deoxygenated hemoglobin, collagen, and lipid. The perspectives of the study include: 1) Improved diagnosis of tendinopathy by assessment of vasculature in the early phase of vessel and nerve ingrowth prior to overt symptoms. 2) Understanding compositional changes that relate to the outcomes of Achilles tendon rupture, to inform improved rehabilitation strategies. 3) Improved understanding of the persistent compositional changes in muscle strain injuries, enabling targeted rehabilitation to reduce recurrence risk.

Our hypotheses were: 1) In both muscle and tendon, exercise will lead to increased hemoglobin contents. 2) Tendinopathic tendons will display greater baseline hemoglobin contents and a greater increase with exercise than the uninjured tendon due to vascular ingrowth. 3) Tendons recovered from a previous tendinopathy will have normal blood contents at baseline but will increase more with exercise due to remaining vessels. 4) Ruptured Achilles tendons will have reduced collagen content. 5) In muscle strain injuries, the muscle will display greater collagen and lipid contents and the aponeurosis will display reduced collagen content.

## 2 Methods

The study was designed as a pilot trial and measurements were conducted over the course of two weeks. The goal of the trial was to evaluate the applicability of MSOT in various musculoskeletal disorders and therefore included a smaller number of participants with several different diagnoses. Due to the condensed period of testing, the participants represent a convenience sample recruited over approximately one month preceding the test period. Participants had been diagnosed with either a chronic (> 3 months) patellar tendinopathy (CPT), an early (< 4 months) Achilles tendinopathy (EAT), a recovered (> 1 year) patellar tendinopathy (RPT), an Achilles tendon rupture (ATR), or a muscle strain injury in the medial gastrocnemius (MSI). The focus for the tendinopathies (CPT, EAT, RPT) was hemoglobin and for the traumatic injuries (ATR, MSI) it was collagen and lipid. The study was conducted in accordance with the Helsinki Declaration and approved by the Danish Medical Research Ethics Committee (#21140002). All participants provided informed, written consent prior to participation. An overview of the participant characteristics can be seen in Table 1.

**Table 1:** Participant characteristics.

Group	Sex [M/F] (Counts)	Age (Years)	Height (cm)	Weight (Kg)	BMI (Kg/m^2^)	Time Since Injury (Months)	Pain Daily Living (NRS)	Satisfaction Daily Living (Likert)
CPT	4/0	31 [22–34]	185 [184–186]	81 [77–90]	24 [22–24]	10 [6–18]	1 [0,1]	4 [3–5]
EAT	4/0	42 [36–48]	187 [179–190]	88 [83–89]	26 [23–27]	2 [1–4]	3 [0–8]	5 [4,5]
RPT	4/0	37 [31–45]	183 [179–185]	85 [76–96]	25 [23–30]	57 [45–58]	0 [0,1]	5 [4,5]
ATR	6/0	44 [38–58]	181 [176–194]	88 [75–104]	25 [23–32]	6 [1–26]	1 [0–3]	3 [1–4]
MSI	4/1	48 [30–70]	174 [168–181]	71 [65–100]	24 [22–31]	6 [1–15]	1 [0–3]	4 [1–5]

CPT: Chronic Patellar Tendinopathy. EAT: Early Achilles Tendinopathy. RPT: Recovered Patellar Tendinopathy. ATR: Achilles Tendon Rupture. MSI: Muscle Strain Injury. M/F: Male/Female. NRS: Numeric Rating Scale (0−10). Likert: Satisfaction scale (1−5). Median [range].

### 2.1 Diagnoses

Participants with a chronic unilateral patellar tendinopathy (CPT) were recruited from an ongoing study on the effects of blood flow restriction on tendinopathy rehabilitation in males (clinicaltrials.gov ID: NCT04550013). Prior to inclusion in the original study, participants were diagnosed with unilateral patellar tendinopathy by a medical doctor, based on clinical symptoms and tendon structure on ultrasound (Doppler, thickening and hypoechoic presence).

Participants with an early unilateral Achilles tendinopathy (EAT) were recruited via online adverts. Participants were diagnosed by an experienced sports physical therapist, based on clinical symptoms (focal pain on palpation, pain during activity) and at least one of the following ultrasound findings: Swelling, presence of a hypoechoic region or power Doppler activity within the tendon.

Participants with a recovered patellar tendinopathy (RPT) were recruited from a previous study [13] on exercise-based tendinopathy rehabilitation in male athletes. Participants had initially been diagnosed according to the same criteria as the chronic participants above and had subsequently undergone a 12-week rehabilitation intervention using slow resistance exercises. At the time of the present study, it was at least one year since the start of the rehabilitation and the included participants reported no clinical symptoms.

Participants with a unilateral Achilles tendon rupture (ATR) were recruited at different timepoints following their injury. Two participants had their rupture >1 year prior and were recruited from an ongoing study (not registered on clinicaltrials.gov). Two participants had their rupture 6 months prior and were recruited from another ongoing study (clinicaltrials.gov ID: NCT04263493). Finally, two participants had a rupture 2–3 months prior and were recruited from the clinic. One of the >1-year participants and the two 6-month participants had received surgery, while the others had received non-surgical treatment.

Participants with a muscle strain injury (MSI) of the medial gastrocnemius were recruited from an ongoing study on muscle function and nutrition (clinicaltrials.gov ID: NCT04100161). The participants had persistent symptoms and displayed structural changes at the gastrocnemius muscle insertion on ultrasound (hypoechoic, curvilinear muscle fascicles and thickened aponeurosis).

### 2.2 Conventional ultrasound

Conventional B-mode and power Doppler ultrasound (Ascendus, Hitachi) was performed prior to the MSOT examination to determine the location of the injury and quantify the Doppler signal. All scans were performed with standardized settings as in previous studies [13]. B-mode images used an 8 cm long probe (EUP-L53L, Hitachi) with 45 mm depth at a central frequency of 10 MHz. Power Doppler was performed with a 3.5 cm long probe (EUP-L75, Hitachi) with 20 mm depth at a central frequency of 10 MHz and pulse repetition frequency of 250 Hz. Doppler was quantified as the total area in mm^2^ with detectable Doppler signal as previously described [13].

### 2.3 Multispectral optoacoustic tomography

All MSOT scans were performed using the MSOT Acuity Echo CE system (iThera Medical GmbH, Munich, Germany). This system is equipped with a probe composed of a tomographic transducer (125° angular coverage and 256 elements) with the following settings: 4 MHz center of frequency, 40 mm×40 mm field of view and a resolution at 10 mm depth of <0.3 mm in-plane and <2.5 mm out-of-plane. The optoacoustic images were acquired using an Optical Parametric Oscillator (OPO) laser with short pulses of <10 ns at a rate of 25 Hz and co-registered with ultrasound images. MSOT data included signals from different wavelengths (700, 730, 760, 800, 850, 910, 930, 950, 980, 1030, 1080, 1100 and 1210 nm), allowing spectral unmixing of adequate chromophores (see Section 2.6). The probe was held still to prevent spatial desynchronization between the recorded wavelengths. Recordings of 5 s duration were performed to maximize the SNR ratio based on per-wavelength averaging. A minimum threshold of 6 frames (based on the provided motion indicator) was chosen as a satisfactory scan acceptance criterion. Moreover, B-mode images were used for orientation and attention was put on placement of the region of interest in the center of the FOV (see details in 3.5). All MSOT scans were acquired by clinical ultrasound experienced researchers without previous experience in MSOT imaging. MSOT hands-on training was performed prior to the study by a representative from the device manufacturer (TS). Initial participant scans were performed by the trained researchers and supervised by the representative to ensure proper image quality.

### 2.4 Scanning procedure

All participants rested for at least 5 min before MSOT data acquisition. The patellar tendon and quadriceps muscle were imaged in a supine position with the knee fully extended. The Achilles tendon, gastrocnemius muscle and aponeurosis were imaged in a prone position with the feet hanging freely over the edge of the examination table.

The tendinopathies (CPT, EAT and RPT) were assessed at rest and after acute exercise (see details in Section 2.5), while traumatic injuries (ATR and MSI) were only assessed at rest in different scan locations. For tendinopathies both muscle and tendon tissue were assessed, for MSI the muscle and aponeurosis were assessed and for ATR only the tendon was assessed. Tendons were always assessed before muscle. For CPT, a time-series of single scans was performed, while the remaining groups had 3 repeated scans at each location and timepoint. To improve accuracy of repeated scans, the initial location of the probe was marked on the skin with a surgical marker. A detailed description and lists of each scan performed and their specific order for each group can be found in the supplementary material (Section S1, Table S1-S4). All scans were performed identically on both legs.

### 2.5 Exercise interventions

An exercise intervention was performed for the CPT, EAT and RPT groups to determine if an exercise response could be measured with MSOT. The ATR and MSI groups were only scanned in the resting state.

The CPT group was first scanned on both sides (injured and healthy) at rest and then following a blood flow restricted exercise performed first on the injured limb followed by MSOT imaging, then repeated on the healthy limb. A hand operated pressure cuff was placed around the upper thigh and inflated to 80 % of the participants arterial occlusion pressure, then they performed knee extensions from 90° to slightly less than full extension in a leg press machine at an external load of 30 % of 1 repetition maximum (1RM). The 1RM and arterial occlusion pressure had been determined previously as part of the study from which the participant was recruited. The exercises consisted of four sets with 45 s of restitution between each set, the first set had 30 repetitions and the following three had 15.

The EAT and RPT groups were scanned on both sides (injured and healthy) at rest and after 10 min of resistance exercises performed first on the injured limb followed by MSOT imaging, then repeated on the healthy limb. The exercises were performed over 10 min as 10 sets each consisting of 30 s of repeated loading (approximately 3 s per repetition) and 30 s restitution. For the EAT group, loading consisted of full plantar flexions in a leg press machine with the knee fully extended against an external load of 30 kg. If the participant felt that the load was insufficient to exert themselves after 5 sets, the load was increased to 40 kg. For the RPT group, loading consisted of knee extensions from 90° to slightly less than full extension in a leg press machine against an external load of 40 kg. Again, if the participant felt that the load was insufficient to exert themselves after 5 sets, the load was increased to 50 kg. For all groups, MSOT imaging was performed as fast as possible after the exercise (∼10 s).

### 2.6 Analyses of optoacoustic data

Approximately 900 scans were collected across all participants and conditions. Data analysis was performed using iLabs software (v.1.3.12b, iThera Medical). The analyzed frames were selected according to the satisfactory scan acceptance criterion (stated in Section 2.3). The regions of interest (ROI) were drawn based on the B-mode images and consisted of two types: one ROI that covers the entire visible tissue under investigation (tendon, muscle or aponeurosis) and one optimized ROI consisting of a smaller circle or rectangle placed within the tissue in a location near the center of the field of view (where illumination and optoacoustic sensitivity is greatest) as has been done previously [44]. Additionally, the optimized ROI was drawn within the region of the injury in a manner that avoids large blood vessels as well as apparent imaging artifacts (e.g. reflections) based on the 700 nm absorbance image. All ROIs were drawn prior to quantification and data analyses. The primary data reported here are extracted from the optimized ROI, additional data from the full tissue regions can be found in the supplementary material.

Using the iLabs software, reconstruction of the raw data was performed by a back projection algorithm (including band-pass filtering and deconvolution). To compensate for light attenuation and improve visualization of signal at depth, a light fluence model was applied. Following reconstruction, a linear regression algorithm was used to spectrally unmix the different chromophores: an estimate of deoxygenated hemoglobin (Hb), an estimate of oxygenated hemoglobin (HbO2), an estimate of collagen (Spectrum C), an estimate of lipids (Spectrum L) and an estimate of water (Spectrum H). In addition to the measured chromophores, MSOT derived total hemoglobin (HbT) and MSOT derived oxygen saturation (mSO2) were also calculated. To reduce the influence of multiple chromophores in the unmixing process, two procedures were used: The first one specific to blood parameters (Hb and HbO2) and using only the lower wavelengths (700–850 nm). The second one including all components (Hb, HbO2, Spectrum C, Spectrum L, and Spectrum H) using all wavelengths (700–1100 nm). From the second unmixing procedure, only Spectrum C and Spectrum L are reported, the other components were included purely as confounders. Quantification within each ROI was limited to pixels with positive signal (due to lack of penetration, the deeper regions of the image had pixels with zero signal) and the mean value of the pixels with positive signal within the ROI is reported for each component.

### 2.7 Statistics

For all the scan procedures involving repeated measurements (all except CPT) the median of the three repeated measures was used for further analyses. For the CPT group, only one scan was performed at each timepoint. As a measure of reproducibility, the coefficient of variation (CV) is reported. The CV was calculated across the repeats for each recording and the median (due to non-normality) of the CV values was determined across all recordings. Power Doppler was compared between the injured and uninjured limb by a non-parametric paired Wilcoxon test and reported as median and inter-quartile range (IQR), due to a non-normal distribution. All other outcomes were analyzed by parametric statistics. The limited sample size hampers a formal normality test, but evaluation of quantile-quantile plots supports the assumption of normality for both hemoglobin, collagen and lipid measures. MSOT outcomes in groups that did not perform exercise (ATR and MSI) were analyzed by a two-way repeated measures (repeated in both factors) mixed model analysis with limb (injured / uninjured) and scan position (sagittal at injury / axial at injury / axial location away from the injury) as factors. For the remaining groups (CPT, EAT and RPT) the same type of two-way repeated measures (repeated in both factors) mixed model analysis was performed with limb (injured / uninjured) and timepoint (pre-exercise / post-exercise / additional timepoints) as factors. Statistical calculations were performed with GraphPad Prism (v.9.5.1). Factors with more than 2 levels (scan position and time) were analyzed by post-hoc tests with multiplicity correction. In the case of scan position all positions were compared using Sídák's correction and for time all time-points were compared to the first time-point with Dunnett's correction.

Due to the pilot nature of the study, it was not powered for significance in specific outcomes, consequently more general observations are also discussed, even if not statistically significant. Significance marks are used according to the following convention: (*) p<0.1, * p<0.05, ** p<0.01, *** p<0.001.

## 3 Results

Basic participant characteristics are presented in Table 1. We included four participants in each of the tendinopathy groups, six participants with Achilles ruptures and five with muscle strain injuries. As expected for this patient population, most participants were middle-aged (median age 42 years) and the more recently injured participants in the EAT group reported more pain. A single participant in the MSI group was substantially older than the rest (70 years) and only one participant was female.

The imaging results presented here are focused on the sagittal scans with data from the optimized ROI and are shown as mean values ±SEM for clarity. Only hemoglobin results are shown for the tendinopathies, and only collagen/lipid for the traumatic injuries. Results and images showing individual datapoints, axial scans, full tissue regions, collagen/lipid for the tendinopathies and hemoglobin for the traumatic injuries can be found in the supplementary material.

### 3.1 Reproducibility

Table 2 shows the CV over repeated scans performed at the same location after removing the probe from the skin and relocating the target site. The results suggest that all hemoglobin associated parameters (HbT, Hb, HbO2 and mSO2) and collagen (Spectrum C) signals have very good reproducibility, which could be explained by a high signal intensity. In contrast, the lipid signal (Spectrum L) showed a 2 to 4-fold greater CV compared to the other parameters. Since lipid was determined from the same images as the other parameters, this difference must be related to the signal quality and might be explained by inherently lower absorbance and greater attenuation by water at the higher wavelength used for detecting lipids (see later discussion).

**Table 2:** Median coefficient of variation across the three repeated scans of the same location for all images. Tendon data from EAT, RPT and ATR groups. Muscle data from EAT, RPT and MSI groups.

	Tendon Sagittal		Tendon Axial		Muscle	
Outcome	Injured	Healthy	Injured	Healthy	Injured	Healthy
HbT	8.0 %	4.1 %	4.7 %	9.1 %	4.6 %	6.6 %
Hb	9.5 %	4.7 %	7.0 %	9.4 %	6.7 %	8.6 %
HbO2	7.2 %	4.7 %	4.9 %	8.6 %	4.9 %	7.6 %
mSO2	2.9 %	2.9 %	4.0 %	5.6 %	1.5 %	2.9 %
Spectrum C	6.5 %	5.1 %	5.6 %	5.6 %	6.7 %	8.5 %
Spectrum L	18.9 %	15.3 %	21.2 %	30.6 %	18.7 %	17.1 %

HbT: Total hemoglobin. Hb: Deoxygenated hemoglobin. HbO2: Oxygenated hemoglobin. mSO2: Oxygen saturation. Spectrum C: Collagen. Spectrum L: Lipids.

### 3.2 Blood parameters

#### 3.2.1 General observations

**Fig. 1:**
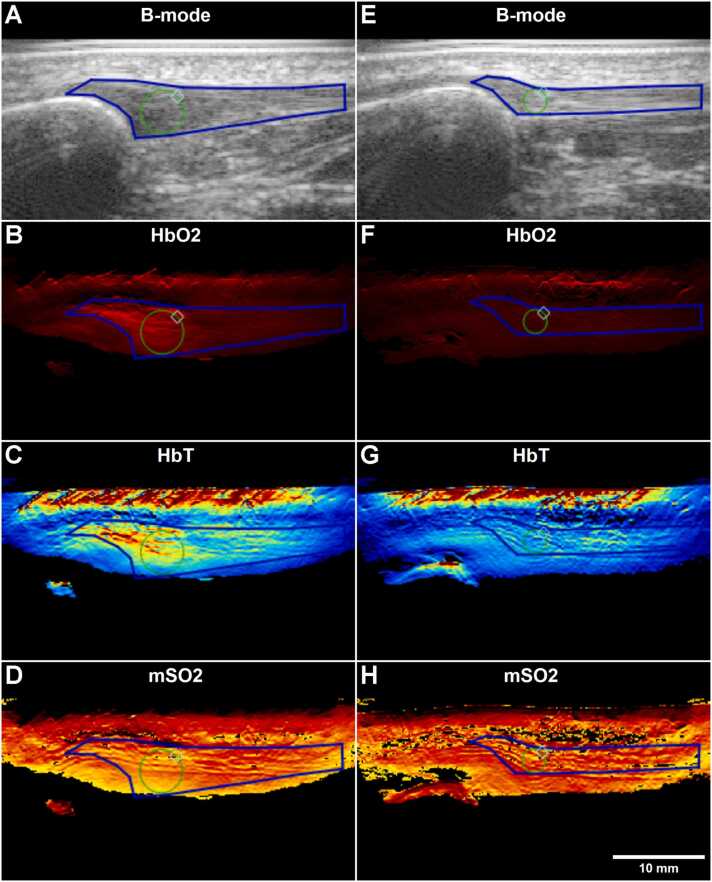
Example of sagittal MSOT images on the injured (A-D) and uninjured (E-F) patellar tendon of a participant with chronic patellar tendinopathy (CPT). A+E) B-mode. B+F) Oxygenated hemoglobin. C+G) Total hemoglobin. D+H) Oxygen saturation. ROIs delineating the entire visible tendon (blue) and the optimized central region (green) are shown.

While the imaging depth at individual wavelengths was generally in the range of 15–20 mm, the maximum depth from which spectrally unmixed data could be obtained in these tissues was around 10–15 mm (Fig. 1). In tendinopathic tendons with observable Doppler, hemoglobin signal was often present in discernable vessel structures of high hemoglobin concentration, while tendinopathic tendons with less Doppler and healthy tendons generally displayed more diffuse signal without discernible vessels (Fig. 1). There was significant hemoglobin signal from the skin as well, but little from the subcutaneous fat or the fat pad underneath the tendon. It should be noted that part of the high skin signal is likely artifactual due to the probe-skin interface and presence of melanin.

#### 3.2.2 Chronic Patellar Tendinopathy (CPT)

**Fig. 2:**
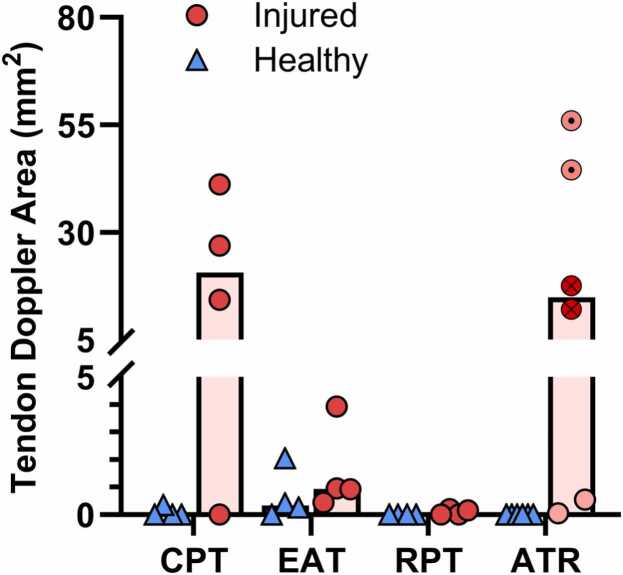
Area of detectable blood flow in tendons determined by power Doppler. CPT: Chronic patellar Tendinopathy, EAT: Early Achilles Tendinopathy, RPT: Recovered Patellar Tendinopathy, ATR: Achilles Tendon Ruptures. For ATR, the datapoints are marked according to duration since injury, cross: 2–3 months, dot: 6 months, unmarked: >1 year.

Power Doppler area was not statistically different between the tendinopathic and contralateral leg, however, 3 of the 4 participants had substantial Doppler signal on the injured side, and the median area was 20.6 mm^2^ (IQR: 3.6–37.6 mm^2^) as expected for a chronic patellar tendinopathy (Fig. 2).

**Fig. 3:**
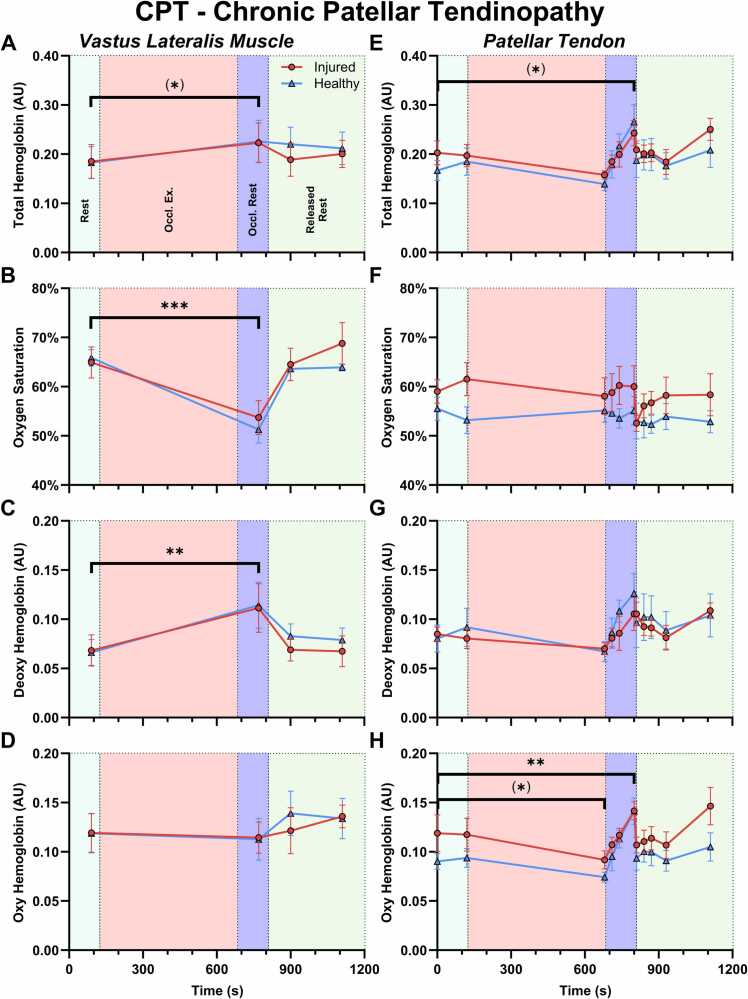
Hemoglobin related MSOT parameters in the vastus lateralis muscle (A-D) and the patellar tendon (E-H) of the chronic patellar tendinopathy participants (CPT) undergoing occlusion exercise. A+E) Total hemoglobin. B+F) Oxygen saturation. C+G) Deoxygenated hemoglobin. D+H) Oxygenated hemoglobin. Measurements were made over 2 minutes at rest (0 s, 90 s and 120 s), immediately after completing the occlusion exercise while remaining occluded (680 s) and with 30 s interval (710 s, 740 s, 770 s and 800 s), and finally immediately after releasing the occlusion (810 s), with 30 s interval (840 s, 870 s, 900 s, 930 s) and 5 min after (1110 s). Measurements were made on the muscle at 90 s, 770 s, 900 s and 1110 s, the rest of the measurements were on tendon. The "Injured" and "Healthy" limb refer to the tendon. * Significant effect of time. Values presented as mean ±SEM.

In the muscle (Fig. 3 A-D), all blood parameters from MSOT were identical between the two limbs at baseline and developed comparably over time. After occlusion exercise, there was a non-significant increase in total hemoglobin (22 %, p=0.06) driven by significantly increased Hb (68 %, p=0.002), and a significant decrease in mSO2 (-20 %, p=0.0002), as expected from the blockage of venous backflow and oxygen consumption during exercise. At 90 s after release of occlusion, deoxygenated blood had been replaced by oxygenated blood leading to a normalization of mSO2 and Hb. Total and oxygenated hemoglobin remained somewhat elevated although not statistically significant, indicating an increased blood volume in the vascular bed. At 300 s after release of occlusion the values remained largely unchanged compared to 90 s after release.

In the tendon (Fig. 3 E-H) there was no statistically significant difference between the injured and uninjured limbs at the baseline, nor were there any differences between the limbs in their development over time. The only numeric, but non-significant, difference was a greater level of HbO2 on the injured side (18 %, p=0.09). Changes over time were mostly not significant, although it should be noted that this in part is due to multiplicity correction for the large number of timepoints. There was a pattern indicating a mostly constant oxygen saturation, meaning that changes in Hb and HbO2 followed the total hemoglobin. The hemoglobin measures displayed non-significant decreases immediately following occlusion exercise (HbT: −20 %, p=0.4; Hb: −17 %, p=0.8; HbO2: −21 %, p=0.09), which was followed by a continuous increase during post-exercise rest with the knee extended while still occluded, reaching a value higher than that at baseline (HbT: 32 %, p=0.05; Hb: 31 %, p=0.2; HbO2: 31 %, p=0.006). Upon release of the occlusion, total hemoglobin rapidly dropped to baseline levels where it remained, at least up to 300 s, at which point there appears to be an increase again.

#### 3.2.3 Early Achilles Tendinopathy (EAT)

Power Doppler area (Fig. 2) was not significantly different between the tendinopathic and contralateral leg, and in contrast to the CPT group, none of the participants had much Doppler signal on the injured side, with a median area of only 0.9 mm^2^ (IQR: 0.6–3.2 mm^2^).

In the muscle, all hemoglobin parameters from MSOT were identical between the two limbs at baseline and did not change with exercise in either limb (Fig. S5 in the supplement).

**Fig. 4:**
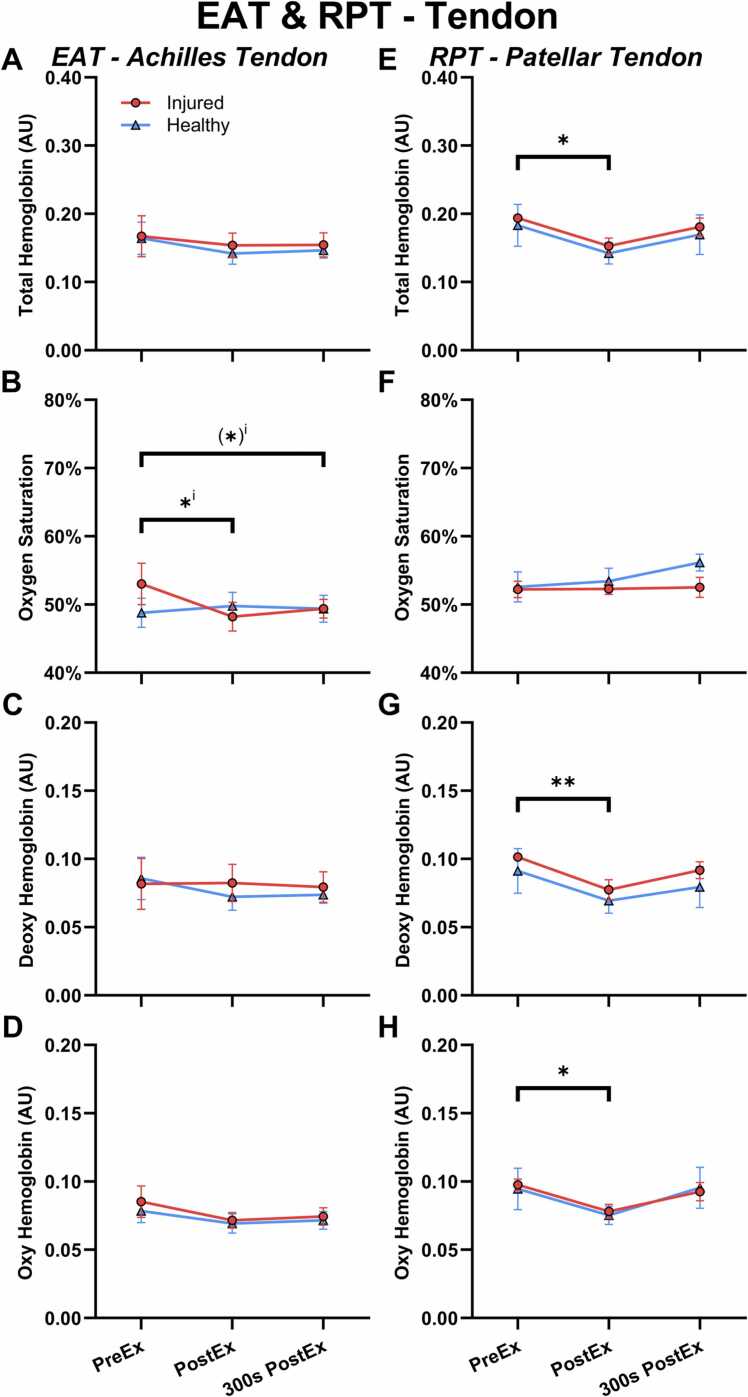
Hemoglobin related MSOT parameters in the Achilles tendon of the early Achilles tendinopathy participants (EAT) (A-D) and the patellar tendon of the recovered patellar tendinopathy (RPT) participants (E-H). A+E) Total hemoglobin. B+F) Oxygen saturation. C+G) Deoxygenated hemoglobin. D+H) Oxygenated hemoglobin. Measurements were made before exercises (PreEx), approximately 30 s after completing the exercises (PostEx) and 300 s after the exercise (300 s PostEx). * Significant effect of time. ^i^ Interaction between time and limb. Values presented as mean ±SEM.

In the tendon (Fig. 4 A-D) there was a significant statistical interaction (time x limb, p=0.006) for the oxygen saturation, displaying a decrease only on the injured limb immediately after exercise (-9 %, p=0.02), which tended to remain over time (300 s, −7 %, p=0.08). For HbT, Hb and HbO2, there were no significant differences but, similar to the CPT group, there was a numerical reduction in hemoglobin immediately after exercise (HbT, −11 %, p=0.5), which remained reduced over time (300 s, −12 %, p=0.4).

#### 3.2.4 Recovered Patellar Tendinopathy (RPT)

Power Doppler area (Fig. 2) was not significantly different between the tendinopathic and contralateral leg, and there was essentially no Doppler signal on the injured side, with a median barely above zero (0.1 mm^2^; IQR: 0.0–0.2 mm^2^).

In the muscle (Fig. S9 in the supplement) there was no differences between the two limbs and the oxygen saturation did not change with exercise. For the hemoglobin measures, there was a reduction after exercise, which was significant for the total and oxygenated hemoglobin (HbT: −26 %, p=0.03; Hb: −20 %, p=0.13; HbO2: −30 %, p=0.04).

The tendon (Fig. 4 E-H) displayed no differences between the limbs, and the oxygen saturation did not change with exercise, but there was a statistically significant decrease in all the hemoglobin measures immediately following exercise (HbT: −22 %, p=0.02; Hb: −24 %, p=0.009; HbO2: −20 %, p=0.03). At 300 s there appeared to be a recovery of the hemoglobin signals back to baseline.

### 3.3 Collagen and lipid

#### 3.3.1 General observations

**Fig. 5:**
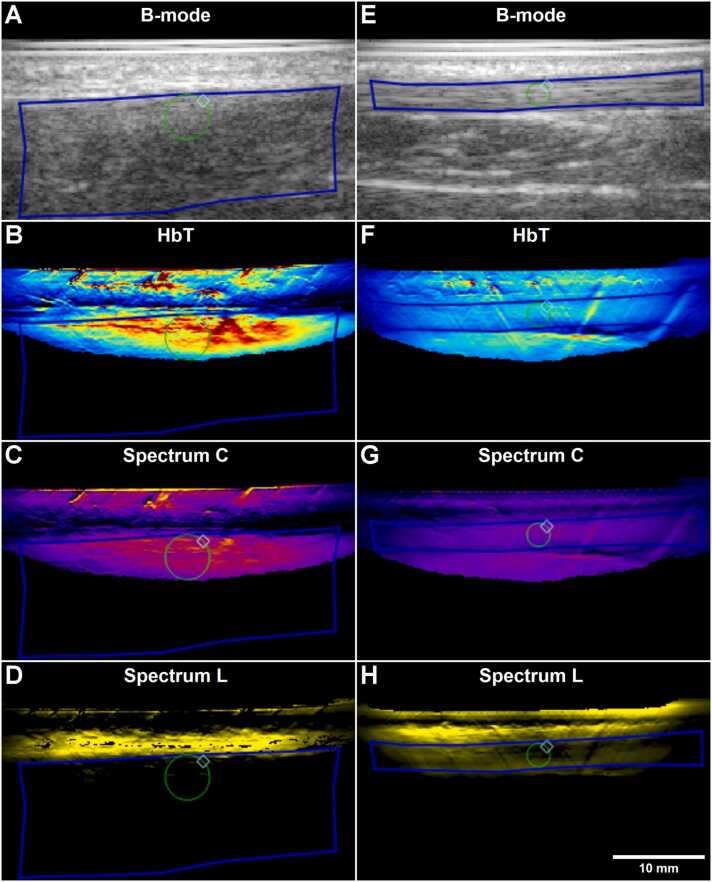
Sagittal MSOT images of an injured (A-D) and an uninjured (E-H) Achilles tendon from the Achilles tendon rupture group (ATR). A+E) B-mode. B+F) Total hemoglobin. C+G) Collagen (Spectrum C). D+H) Lipid (Spectrum L). ROIs delineating the entire visible tendon (blue) and the optimized central region (green) are shown.

The depth from which sufficient collagen and lipid signal could be obtained was less than for the blood parameters, which resulted in substantially less signal in the extracted parameters, especially for lipid (Fig. 5, Fig. 7). Typically, there was strong lipid signal from the skin, but generally very little signal was present in the deeper structures. Collagen signal was generally also high at the skin surface with little signal in the subcutaneous region, but unlike lipid, there was more signal from the deeper regions. Surprisingly, the collagen signal of the tendon in general did not stand out substantially from the surrounding tissue, as should be expected considering the high collagen content (60–90 % of dry mass) of tendon tissue.

#### 3.3.2 Achilles Tendon Rupture (ATR)

**Fig. 6:**
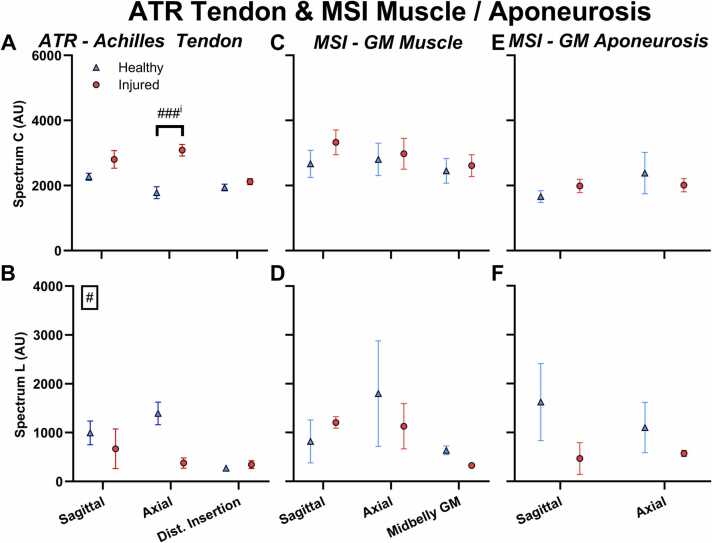
Tissue related MSOT parameters in the Achilles tendon of the Achilles tendon rupture participants (ATR) (A-B) and the muscle (C-D) and aponeurosis (E-F) of the medial gastrocnemius (GM) muscle in strain injury participants (MSI). A+C+E) Collagen (Spectrum C). B+D+F) Lipids (Spectrum L). Scans were performed at the injury site (or the same location in the healthy limb) in both the sagittal and axial plane, and at a site away from the injury (distal insertion of the Achilles (Dist. Insertion) or the midbelly of the gastrocnemius muscle (Midbelly GM)). Values presented as mean ±SEM.

For collagen, the injured tendon had greater signal than the healthy contralateral tendon in the region of the tendon where the rupture had occurred (Fig. 6 A). This was the case for both the sagittal and axial scans but was only statistically significant for the axial. Axial scans further away from the rupture, at a distal site just above the calcaneal insertion, did not display greater collagen signal on the injured tendon (sagittal: 23 %, p=0.1; axial: 73 %, p<0.0001; distal: 9 %, p=0.9). The presence of a significant statistical interaction (limb x scan location, p=0.01) indicated that the difference between the limbs was greater at the rupture site than the distal site.

The opposite pattern was observed for the lipid signal (Fig. 6 B), with lower values in the injured compared to the healthy tendon. The model displayed a main effect of injury (-46 %, p=0.04), indicating that the lower lipid signal was present across all the scan locations, including the distal site.

#### 3.3.3 Muscle Strain Injury (MSI)

For the muscle strain injury, the regions of interest in the muscle and the aponeurosis were fairly deep in the image and as a result, several of the participants showed no lipid signal in these regions (Fig. 6 D), leading to a very small sample size (N=2) (see Fig. S16 in the supplement). For this reason, we do not believe that these results can be meaningfully interpreted. No statistical differences were observed (Fig. 6 D+F).

**Fig. 7:**
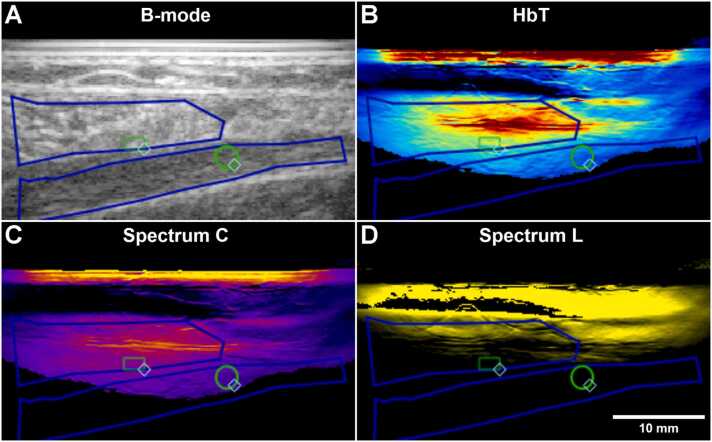
Sagittal MSOT images of the medial gastrocnemius muscle (upper ROI) and aponeurosis (lower ROI) on the injured side of a muscle strain injury participant (MSI). A) B-mode. B) Total hemoglobin. C) Collagen (Spectrum C). D) Lipid (Spectrum L). ROIs delineating the entire visible tendon (blue) and the optimized central region (green) are shown. Note that the horizontal lines with high hemoglobin and collagen signal in the middle of the muscle are likely artefacts due to reflection of the high skin signal in the overlying muscle fascia.

For collagen, most of the participants had sufficient signal to analyze, at least in the muscle and the superficial parts of the aponeurosis (Fig. 7 C). On the aponeurosis side, no clear pattern was observed, and no significant differences were present (Fig. 6 E). On the muscle side, there were no statistically significant differences between the healthy and injured limb either.

## 4 Discussion

Overall, measures of hemoglobin had high reproducibility and displayed systematic changes in musculoskeletal tissues in response to exercise on both the injured and healthy limb, supporting the applicability of MSOT to assess these parameters in the lower limb musculoskeletal tissues of adults. Interestingly, we did not demonstrate any major differences in hemoglobin contents between injured and healthy tendons. In contrast to hemoglobin, collagen and lipid contents estimated with MSOT were less consistent in muscle and tendon from adults, due to limited signal at the depths of the present tissues.

### 4.1 Blood parameters

We included measurements on healthy muscle tissue along with the tendon measurements as a control since more is known about blood perfusion and oxygenation in muscle than tendon. In the CPT group where blood-flow restricted exercises were performed, the vascular response of the muscle followed the expected pattern, indicating that MSOT is sufficiently accurate to measure exercise related changes. This is also consistent with a recent publication on forearm muscles using MSOT during blood flow restriction [40]. Free-flow exercises in the EAT and RPT groups did not increase muscle perfusion or oxygenation. This is in contrast to the acute increase in hemoglobin reported with MSOT after exercise of the forearm [45]. The reason for this difference could be the moderate intensity of exercises in the present study.

The present exercises were designed to mechanically load the tendon rather than aerobically challenging the muscle, and while no effect was seen on muscle there were changes in the tendon, with an acute (although not statistically significant) reduction in total hemoglobin immediately after exercise in all three groups (CPT: −20 %, EAT: −11 % and RPT: −22 %). These results did not match previously published studies using different methods (contrast enhanced ultrasound, xenon washout, NIRS), which reported increased blood flow in tendon after exercise [19], [46], [47]. However, one study using both xenon washout and NIRS reported increased flow with concomitant reduction in total hemoglobin [46]. It is possible that increased flow is not associated with an increased concentration of hemoglobin in tendon. If the blood flows faster through the same volume of vessels, flow sensitive methods like Doppler will register a rise, but the hemoglobin concentration in the tendon at any given time may be constant. The independence of flow rate makes hemoglobin measures especially useful for assessing microvasculature where flow rates are very low. This advantage is apparent in the present data, where systematic changes in hemoglobin are detected with blood flow restriction and exercises, even in healthy tendons where no flow is detectable with Doppler. Surprisingly, there were no baseline differences in hemoglobin measures between the tendinopathic and contralateral limb in the chronic participants (CPT), despite substantial differences in Doppler. This suggests that a greater Doppler signal in injured tendon [5], [48] mainly represents an increase in the flow velocity rather than an increase in the total volume of blood present. We believe that dedicated studies to address these hypotheses could provide novel insight into the vasculature of both injured and healthy tendons.

The timing of measurements also plays a role, and a previous study using Doppler in tendinopathy patients reported a reduction in blood flow immediately after exercise [49], in agreement with the present data. We consider the most likely mechanism for reduced tendon flow immediately after exercise to be direct constriction of blood vessels by mechanical loading of the tendon, which over a period of time "squeezes" some of the remaining blood out of the tissue. This idea is supported by MRI studies reporting reduced tendon volume and water content following mechanical loading [50]. Following the acute decrease in hemoglobin concentration after exercise, there was an increase compared to the post-exercise value in both the CPT (64 %) and RPT (19 %) groups. For the CPT group, total hemoglobin continued to rise during the occluded resting phase and reached greater levels than at baseline. There was no clear attenuation of this rise at 120 s, suggesting that even higher levels may have been reached with continued occlusion. Because the hemoglobin level drops back to baseline almost immediately upon release of the occlusion, we interpret the rise to be a result of accumulating blood due to the venous occlusion, rather than an effect of vasodilation. This is also supported by the behavior of the RPT group, in which total hemoglobin recovers but does not exceed the baseline value after 300 s.

### 4.2 Collagen and lipids

The extraction of deoxygenated and oxygenated hemoglobin from near-infrared spectroscopy is well established [21], [25]. Extraction of collagen and lipid is less well established and made more difficult by lower inherent absorbance of the targets and relying on higher wavelengths where increased water absorbance leads to inferior penetration. Very limited lipid signal could be obtained and while more collagen signal was apparent the localization and appearance in many cases deviated from what we expected, with the collagen-rich tendon not standing out from the surrounding tissues (Fig. 5). In addition, the collagen signal pattern in the images was similar to the hemoglobin signal both visually and quantitatively even when comparing values before and after exercise (Fig. S1+S2 in the supplement) where it is not physiologically reasonable for the collagen content to change. This indicates a possible "bleed through" of the hemoglobin signal. The spectral unmixing for collagen accounted for both oxygenated and deoxygenated hemoglobin, but we speculate that spectral coloring due to preferential absorption of high wavelengths in the skin and subcutis skewed the spectral unmixing in deeper regions resulting in the hemoglobin signal getting interpreted as collagen [51]. Spectral coloring is also a major challenge in the quantification of oxygen saturation and can lead to significant errors in the absolute measures of saturation [52]. However, it is worth noting that within-subject changes over time at the same scan location (as in Fig. 3), may be less affected since the spectral coloring induced by absorbance from the surrounding tissue will be constant. Due to these uncertainties in the reliability of the collagen and lipid signal, we refrain from attempting a physiological interpretation of the observations.

The present difficulties in extracting a reliable collagen signal are somewhat in contrast to previous reports where a convincing collagen signal could be obtained in children with muscular dystrophy and spinal muscular atrophy as well as collagen-containing phantoms [31], [39]. The reason for this difference is likely related to the greater depth of the tissues and thicker (more absorbing) skin and subcutaneous layers in the present adult participants.

### 4.3 Limitations

Due to the exploratory nature of the study, the sample sizes in each group were too small to make any definitive conclusions and several of the observations discussed were not statistically significant. The physiological interpretation of our results should therefore only be considered hypothesis generating. Related to the small sample size, only a single female was included. A priori, sex was not part of the inclusion criteria, but because two of the studies we recruited from only included males and due to the greater prevalence of these injuries in males the population ended up skewed.

While reproducibility on repeated scans was generally good, and the use of skin markings helped relocate the same scan site, it cannot be guaranteed that the exact same location was imaged over the entire scan session, in particular before and after exercise. Some artifacts can be present in MSOT images, most notably reflections that occur when signal from tissue with high absorption (such as the skin) is reflected from a lower lying structure giving rise to a false mirror image. To reduce their impact, smaller ROIs were placed in locations visibly free of such artifacts, however, some artifactual signal could remain in our data. The subjective placement of these ROIs is an important limitation to the quantification, however, results for the entire tissue ROI presented in the supplement, generally agrees with those of the ROI, indicating that ROI placement did not bias the results.

## 5 Conclusion

These preliminary results indicate that multispectral optoacoustic tomography can provide valuable information on hemoglobin-related parameters in superficial tendons and muscles of adults in relation to sports injuries. Importantly, this data is complimentary to that obtained from Doppler ultrasound because it is independent of flow. Use of the method for determining hemoglobin in deeper lying aponeuroses and muscles in adults remains challenging due to limited penetration.

In our hands, the method was presently unable to provide reliable estimates of collagen and lipid in the musculoskeletal tissues of adults.

Regarding our original hypotheses, free-flow exercises at moderate intensity had little effect on muscle blood content but tendons displayed acute reductions with no apparent subsequent hyperemia. Occluded exercises induced measurable increases in deoxygenated blood in muscle and resulted in acute reductions of blood in tendon with subsequent hyperemia while occlusion was maintained. Tendinopathic and uninjured tendons did not display any differences in blood content at baseline or during exercise. The ruptured Achilles tendons had greater hemoglobin content, but collagen and lipid content could not be interpreted, which was also the case for the muscle strain injuries.

## Funding

The study received no specific funding. The Institute of Sports Medicine Copenhagen received support from the 10.13039/501100003554Lundbeck Foundation [R198–2015-207]; 10.13039/501100009708Novo Nordisk Fonden [NNF18OC0052371]; and the Nordea Foundation - Center for Healthy Aging. The funding bodies had no influence on the study.

## CRediT authorship contribution statement

**Monika L. Bayer:** Writing – review & editing, Resources, Investigation, Conceptualization. **Rikke Hoeffner:** Writing – review & editing, Resources, Investigation, Conceptualization. **Christian Couppé:** Writing – review & editing, Resources, Investigation, Conceptualization. **Rene B. Svensson:** Writing – review & editing, Writing – original draft, Visualization, Methodology, Investigation, Formal analysis, Data curation, Conceptualization. **Michael Kjaer:** Writing – review & editing, Resources, Funding acquisition, Conceptualization. **S. Peter Magnusson:** Writing – review & editing, Resources, Project administration, Funding acquisition, Conceptualization. **Charlène Reichl:** Writing – review & editing, Visualization, Methodology, Formal analysis, Data curation. **Mikkel H. Hjortshoej:** Writing – review & editing, Resources, Investigation, Conceptualization. **Anne-Sofie Agergaard:** Writing – review & editing, Resources, Project administration, Methodology, Investigation, Formal analysis, Data curation, Conceptualization. **Thomas Sardella:** Writing – review & editing, Visualization, Methodology, Formal analysis, Data curation.

## Declaration of Competing Interest

The authors declare the following financial interests/personal relationships which may be considered as potential competing interests: Thomas Sardella reports a relationship with iThera Medical GmbH that includes: employment. Charlene Reichl reports a relationship with iThera Medical GmbH that includes: employment. If there are other authors, they declare that they have no known competing financial interests or personal relationships that could have appeared to influence the work reported in this paper.

## Data Availability

Data will be made available on request.
