# Supplementary material for: Application of multispectral optoacoustic tomography for lower limb musculoskeletal sports injuries in adults
Source: Photoacoustics. 2024 Oct 9;40:100656. doi: 10.1016/j.pacs.2024.100656 (PMC11866168; doi:10.1016/j.pacs.2024.100656)
Supplement: Supplementary file 1 — Supplementary material [file mmc1.pdf]

Supplementary material for:

Application of Multispectral Optoacoustic Tomography for Lower Limb Musculoskeletal Sports Injuries in Adults

Rene B. Svensson[a,b*], Anne-Sofie Agergaard[a,b,c], Thomas Sardella[d], Charlène Reichl[d], Mikkel H. Hjortshoej[a,b,c,e], Monika L. Bayer[a,b], Rikke Hoeffner[a,b,c], Christian Couppé[a,b], Michael Kjaer[a,b], S. Peter Magnusson[a,b,c]

[a] Institute of Sports Medicine Copenhagen, Department of Orthopedic Surgery, Copenhagen University Hospital - Bispebjerg and Frederiksberg, Copenhagen, Denmark

[b] Center for Healthy Aging, Department of Clinical Medicine, Faculty of Health and Medical Sciences, University of Copenhagen, Copenhagen, Denmark.

[c] Department of Physical and Occupational Therapy, Copenhagen University Hospital - Bispebjerg and Frederiksberg, Copenhagen, Denmark

[d] iThera Medical GmbH, Munich, Germany

[e] Centre for Health and Rehabilitation, University College Absalon, Slagelse, Denmark

*Corresponding author: Rene B. Svensson (rene.svensson@sund.ku.dk). Institute of Sports Medicine Copenhagen, Bispebjerg Hospital Bld. 8, Nielsine Nielsens Vej 11, 2400 Copenhagen, Denmark.

Abbreviations:
CPT: Chronic Patellar Tendinopathy
EAT: Early Achilles Tendinopathy
RPT: Recovered Patellar Tendinopathy
ATR: Achilles Tendon Rupture
MSI: Muscle Strain Injury
ROI: Region Of Interest
Ex: Exercise (resistance)
Occl: Occlusion

Contents

S1 Scanning procedures 3
Supplementary Tables 4
Table S1. CPT scan order 4
Table S2. EAT and RPT scan order 5
Table S3. ATR scan order 5
Table S4. MSI scan order 5
Supplementary Figures 6
CPT 6
Blood – ROI 6
Collagen, lipid, water – ROI 7
Blood – Whole 8
Collagen, lipid, water – Whole 9
EAT 10
Blood – ROI 10
Collagen, lipid, water – ROI 11
Blood – Whole 12
Collagen, lipid, water – Whole 13
RPT 14
Blood – ROI 14
Collagen, lipid, water – ROI 15
Blood – Whole 16
Collagen, lipid, water – Whole 17
ATR 18
Blood – ROI and Whole 18
Collagen, lipid and water – ROI and Whole 19
MSI 20
Blood – ROI 20
Collagen, lipid, water – ROI 21
Blood – Whole 22
Collagen, lipid, water – Whole 23
MSOT image examples 24
CPT – Injured – Collagen, lipid – Sagittal 24
CPT – Injured – Blood – Axial 25
CPT – Injured – Collagen, lipid – Axial 26
CPT – Healthy – Collagen, lipid – Sagittal 27
CPT – Healthy – Blood – Axial 28
CPT – Healthy – Collagen, lipid – Axial 29
ATR – Inured – Blood – Sagittal 30
ATR – Injured – Collagen, lipid – Axial 31
ATR – Injured – Blood – Axial 32
ATR – Healthy – Blood – Sagittal 33
ATR – Healthy – Collagen, lipid – Axial 34
ATR – Healthy – Blood – Axial 35
MSI – Inured – Blood – Sagittal 36
MSI – Injured – Collagen, lipid – Axial 37
MSI – Injured – Blood – Axial 38

S1 Scanning procedures

Lists of scan locations and the order of scans can be found in Tables S1-4.

CPT: Sagittal scans of the injury location on the patellar tendon were performed before the occlusion exercise, immediately after the exercise while maintaining occlusion for 2 min, and immediately after releasing the occlusion, continuing for up to 5 min after release. Single scans were repeated with regular intervals to determine the post-exercise and post-release response. Additionally, scans on the vastus lateralis muscle 15 cm proximal to the patella were made before the occlusion exercise, 90 s after the exercise, 90 s and 5 min after releasing the occlusion.

EAT: Sagittal and axial scans at the injury location as well as axial scans of muscle at the gastrocnemius mid-belly. Each scan location was repeated 3 times. Scans were performed at rest before the exercise, immediately after the exercise and for the tendon also 5 min after the exercise.

RPT: Sagittal and axial scans at the injury location as well as axial scans of the vastus lateralis muscle 15 cm proximal to the patella. Each scan location was repeated 3 times. Scans were performed at rest before the exercise, immediately after the exercise and for the tendon also 5 min after the exercise.

ATR: Sagittal and axial scans of the rupture site (on the healthy limb at a location the same distance proximal to calcaneus as the rupture site on the injured limb) as well as axial scans at a site distal to the rupture just above the calcaneus. Sagittal scans at this distal site were not performed due to the calcaneus causing poor probe contact. Each scan location was repeated 3 times.

MSI: Sagittal and axial scans at the injury location (near the distal insertion of gastrocnemius medialis (GM) on the Achilles tendon and the same anatomical site on the healthy limb) as well as axial scans of normal muscle at the gastrocnemius mid-belly. Each scan location was repeated 3 times.

Supplementary Tables

Table S1. CPT scan order
(all sagittal, no repeated scans)

**Timepoint**	**Side**	**Occlusion State**	**Tissue**
0s Pre	Injured	None	Tendon
90s Pre	Injured	None	Muscle
120s Pre	Injured	None	Tendon
0s Pre	Uninjured	None	Tendon
90s Pre	Uninjured	None	Muscle
120s Pre	Uninjured	None	Tendon
~8 min occlusion exercise on injured side			
0s PostOcclEx	Injured	Occluded	Tendon
30s PostOcclEx	Injured	Occluded	Tendon
60s PostOcclEx	Injured	Occluded	Tendon
90s PostOcclEx	Injured	Occluded	Muscle
120s PostOcclEx	Injured	Occluded	Tendon
Occlusion cuff released			
0s PostRelease	Injured	Released	Tendon
30s PostRelease	Injured	Released	Tendon
60s PostRelease	Injured	Released	Tendon
90s PostRelease	Injured	Released	Muscle
120s PostRelease	Injured	Released	Tendon
300s PostRelease	Injured	Released	Tendon
300s PostRelease	Injured	Released	Muscle
~8 min occlusion exercise on uninjured side			
0s PostOcclEx	Uninjured	Occluded	Tendon
30s PostOcclEx	Uninjured	Occluded	Tendon
60s PostOcclEx	Uninjured	Occluded	Tendon
90s PostOcclEx	Uninjured	Occluded	Muscle
120s PostOcclEx	Uninjured	Occluded	Tendon
Occlusion cuff released			
0s PostRelease	Uninjured	Released	Tendon
30s PostRelease	Uninjured	Released	Tendon
60s PostRelease	Uninjured	Released	Tendon
90s PostRelease	Uninjured	Released	Muscle
120s PostRelease	Uninjured	Released	Tendon
300s PostRelease	Uninjured	Released	Tendon
300s PostRelease	Uninjured	Released	Muscle

Table S2. EAT and RPT scan order

Timepoint	Side	Orientation	Tissue	Repeats
PreEx	Injured	Sagittal	Tendon	3
PreEx	Injured	Axial	Tendon	3
PreEx	Injured	Axial	Muscle	3
PreEx	Uninjured	Sagittal	Tendon	3
PreEx	Uninjured	Axial	Tendon	3
PreEx	Uninjured	Axial	Muscle	3
10 min resistance exercise on injured side				
PostEx	Injured	Sagittal	Tendon	3
PostEx	Injured	Axial	Tendon	3
PostEx	Injured	Axial	Muscle	3
300s PostEx	Injured	Sagittal	Tendon	3
300s PostEx	Injured	Axial	Tendon	3
10 min resistance exercise on uninjured side				
PostEx	Uninjured	Sagittal	Tendon	3
PostEx	Uninjured	Axial	Tendon	3
PostEx	Uninjured	Axial	Muscle	3
300s PostEx	Uninjured	Sagittal	Tendon	3
300s PostEx	Uninjured	Axial	Tendon	3

Table S3. ATR scan order

Side	Orientation	Tissue	Repeats
Injured	Sagittal	Tendon	3
Injured	Axial	Tendon	3
Injured	Axial	Distal Tendon	3
Uninjured	Sagittal	Tendon	3
Uninjured	Axial	Tendon	3
Uninjured	Axial	Distal Tendon	3

Table S4. MSI scan order

Side	Orientation	Tissue	Repeats
Injured	Sagittal	Muscle + Aponeurosis	3
Injured	Axial	Muscle + Aponeurosis	3
Injured	Axial	Muscle Midbelly	3
Uninjured	Sagittal	Muscle + Aponeurosis	3
Uninjured	Axial	Muscle + Aponeurosis	3
Uninjured	Axial	Muscle Midbelly	3

Supplementary Figures

CPT

Blood – ROI

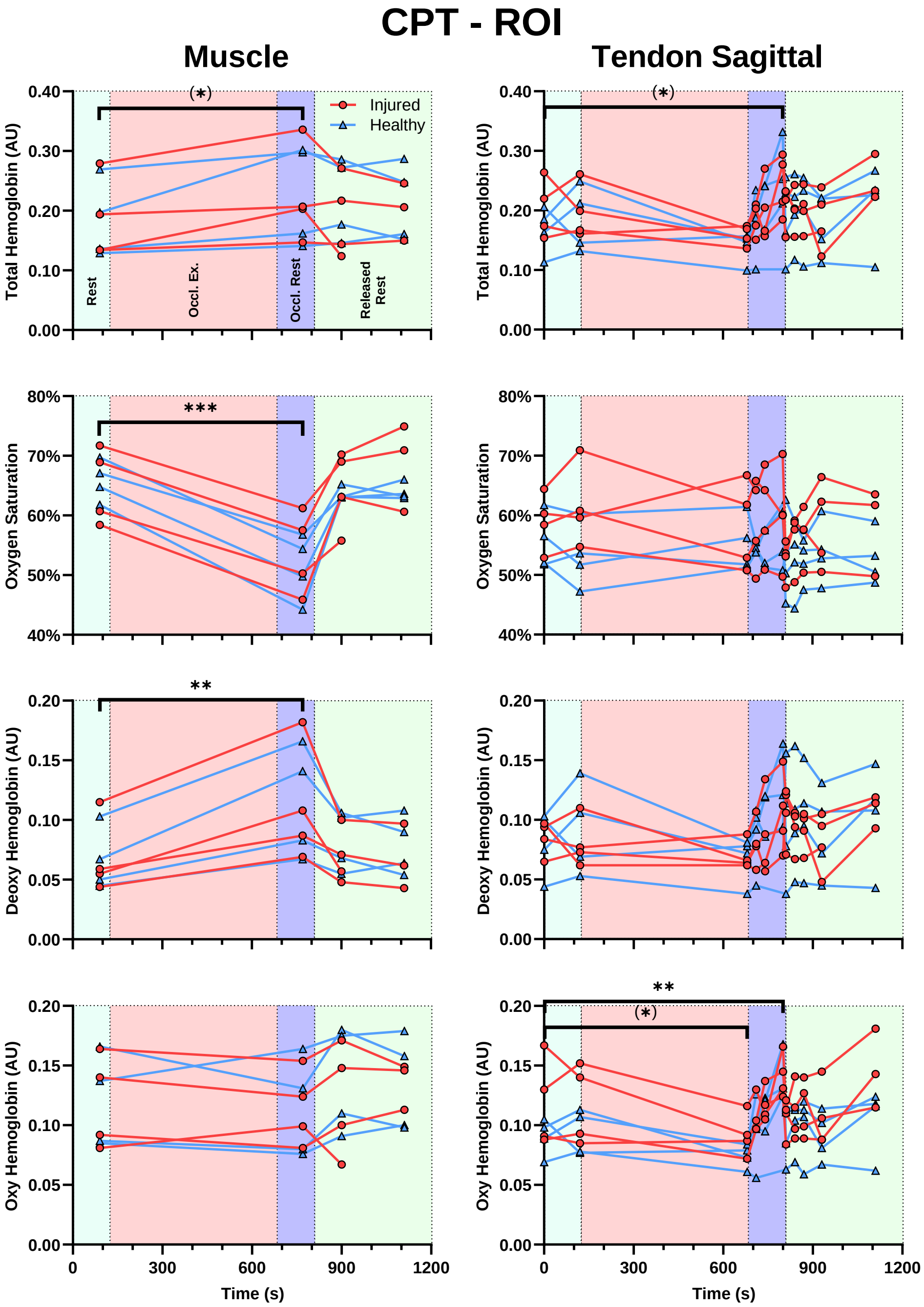

Figure S1. Individual datapoints for blood related MSOT outcomes measured in a smaller optimized ROI in the muscle and tendon of the CPT group across the occlusion exercise intervention. * Significant effect of time. (*) 0.05<p<0.1.

Collagen, lipid, water – ROI

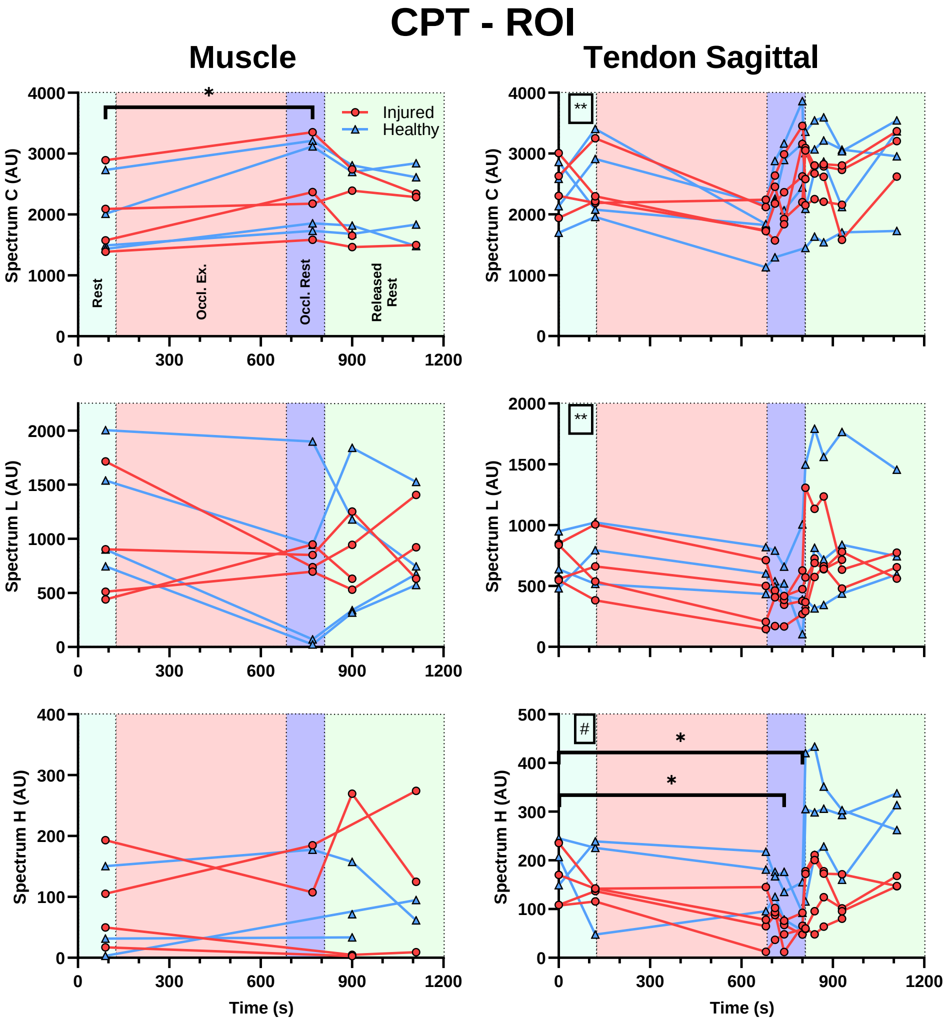

Figure S2. Individual datapoints for collagen (spectrum C), lipid (spectrum L) and water (spectrum H) related MSOT outcomes measured in a smaller optimized ROI in the muscle and tendon of the CPT group across the occlusion exercise intervention. * Significant effect of time. # Significant effect of side (injured/healthy). Significance markers placed in a box indicate a main effect not localized to a specific timepoint.

Blood – Whole

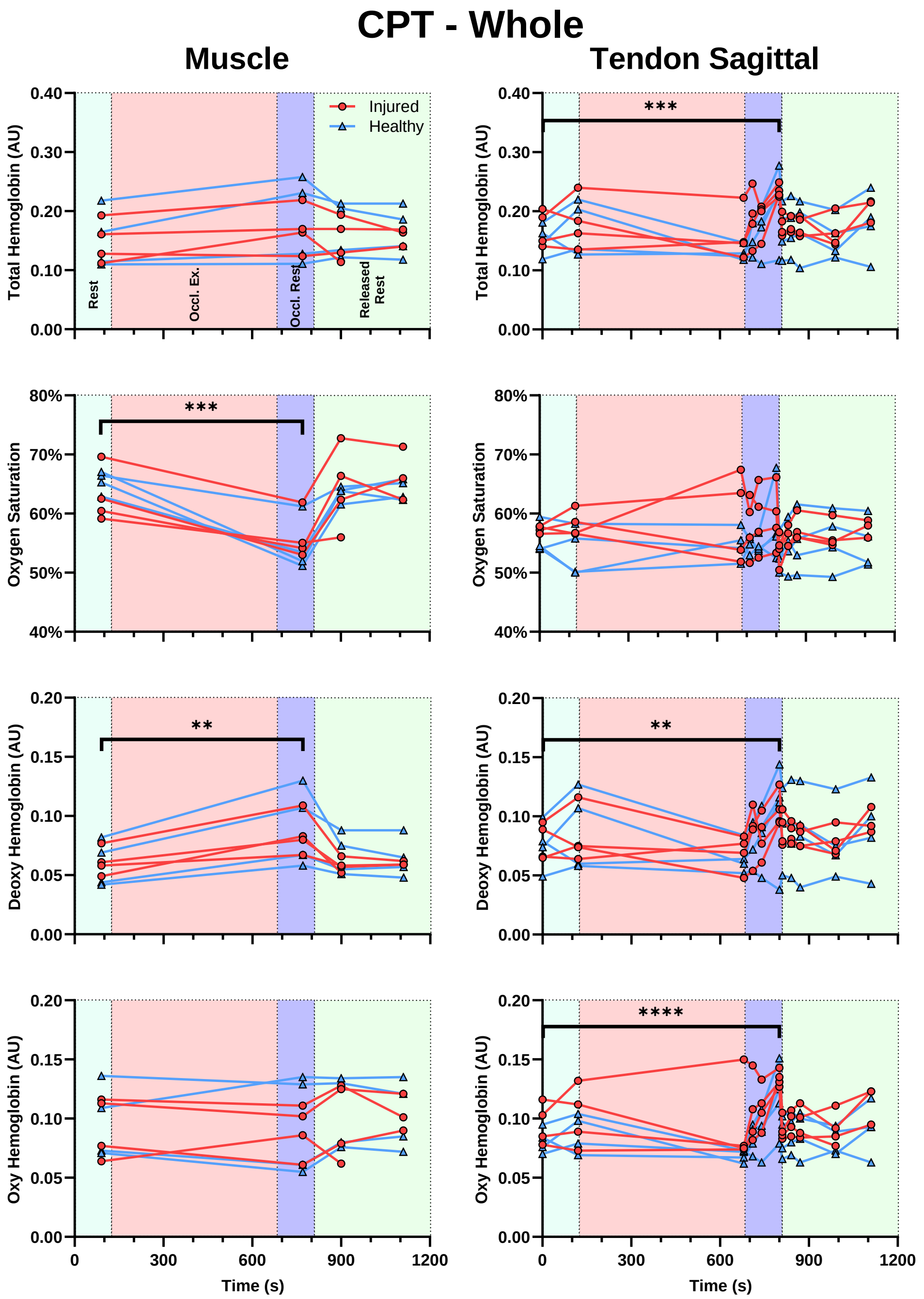

Figure S3. Individual datapoints for blood related MSOT outcomes measured in the whole tissue region of the muscle and tendon in the CPT group across the occlusion exercise intervention. * Significant effect of time.

Collagen, lipid, water – Whole

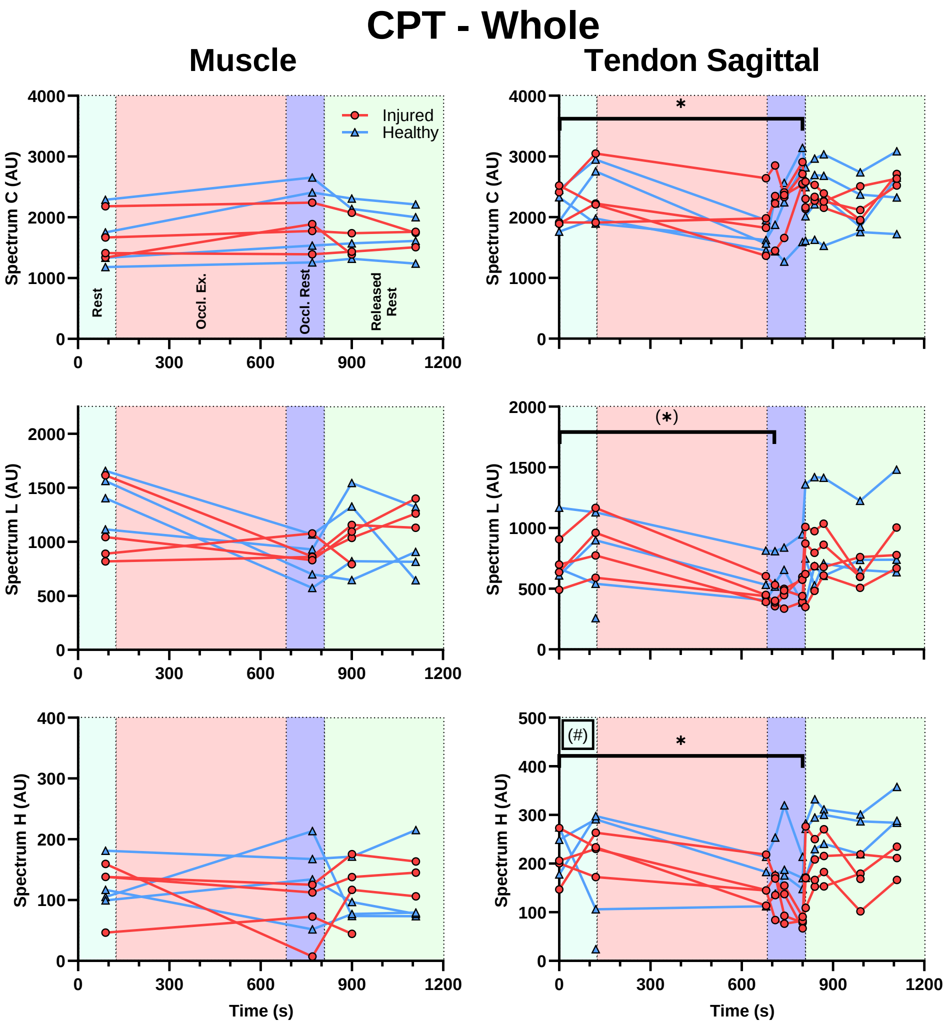

Figure S4. Individual datapoints for collagen (spectrum C), lipid (spectrum L) and water (spectrum H) related MSOT outcomes measured in the whole tissue region of the muscle and tendon in the CPT group across the occlusion exercise intervention. * Significant effect of time. # Significant effect of side (injured/healthy). (*)/(#) $0.05<p<0.1$. Significance markers placed in a box indicate a main effect not localized to a specific timepoint.

EAT

Blood – ROI

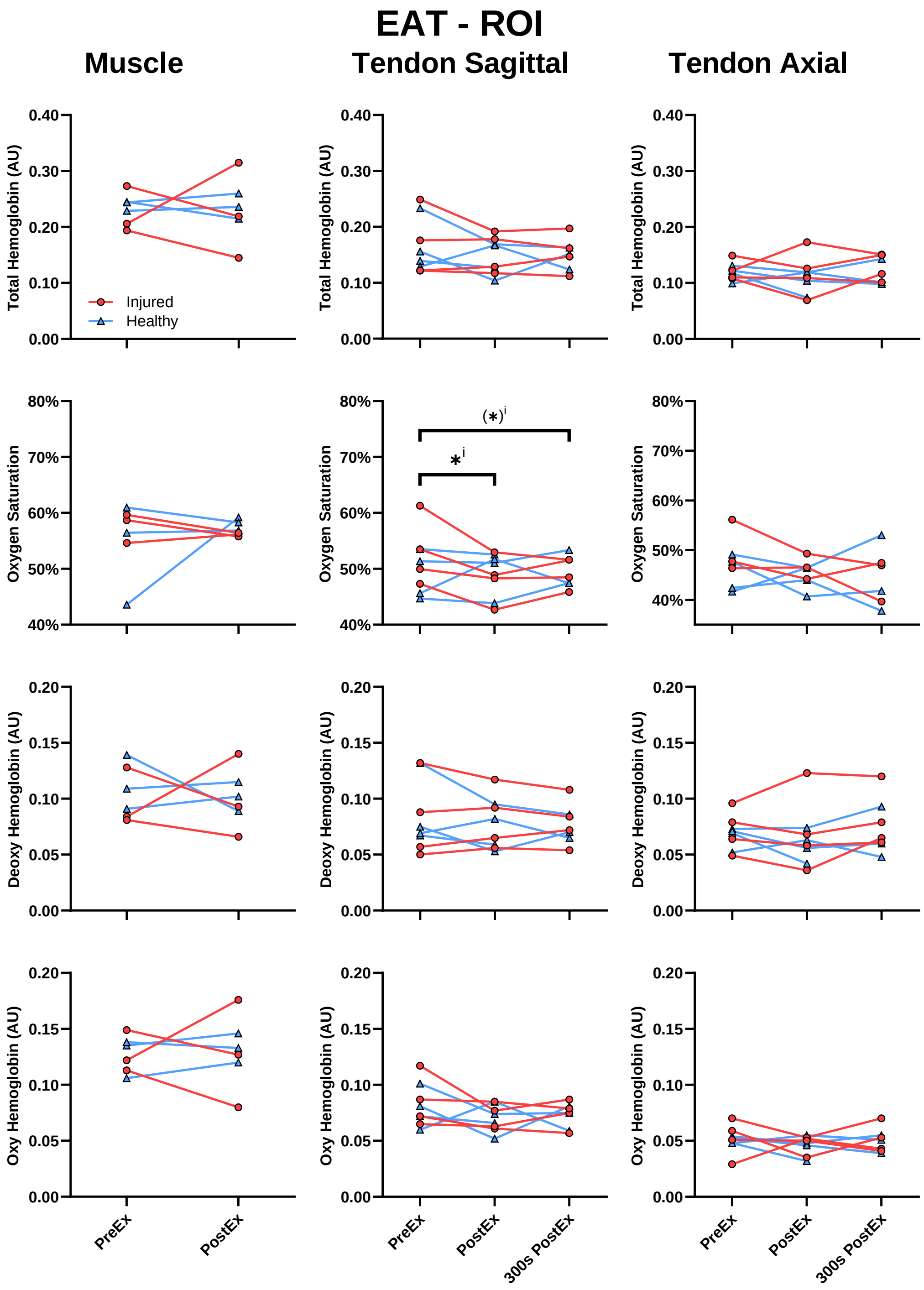

Figure S5. Individual datapoints for blood related MSOT outcomes measured in a smaller optimized ROI in the muscle and tendon of the EAT group, with the tendon measured both on sagittal and axial scans. * Significant effect of time. [i] Significant interaction between time and side.

Collagen, lipid, water – ROI

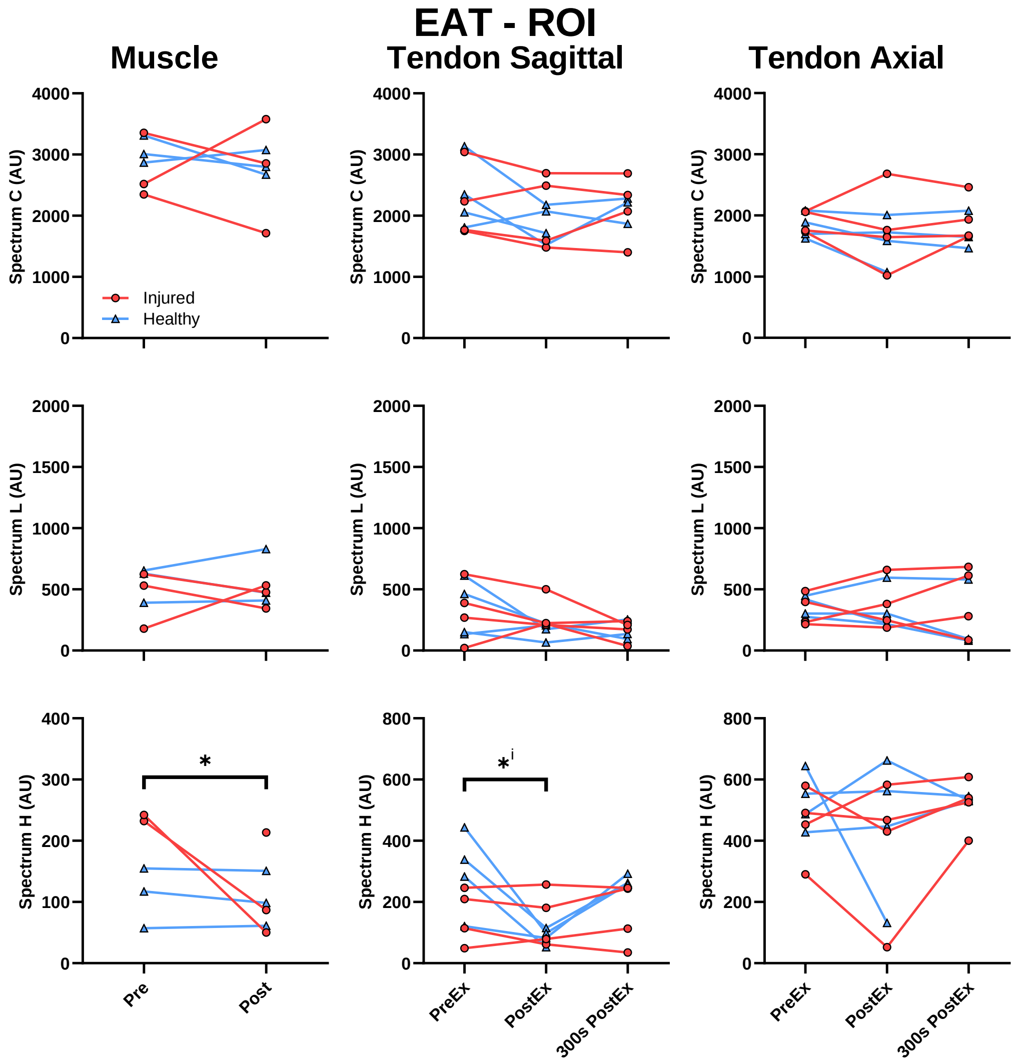

Figure S6. Individual datapoints for collagen (spectrum C), lipid (spectrum L) and water (spectrum H) related MSOT outcomes measured in a smaller optimized ROI in the muscle and tendon of the EAT group, with the tendon measured both on sagittal and axial scans. * Significant effect of time. [i] Significant interaction between time and side.

Blood – Whole

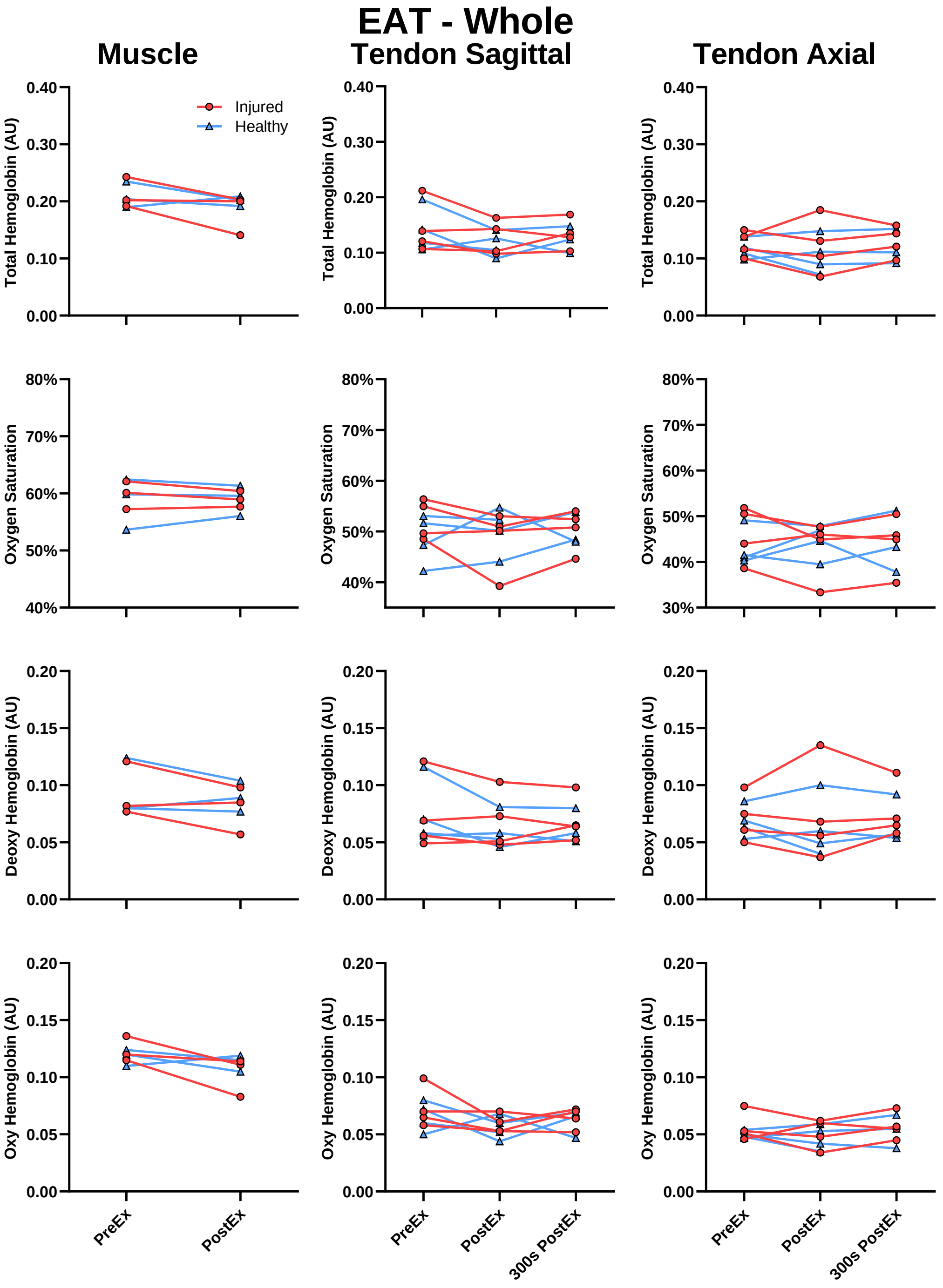

Figure S7. Individual datapoints for blood related MSOT outcomes measured in the whole tissue region of the muscle and tendon in the EAT group, with the tendon measured both on sagittal and axial scans.

Collagen, lipid, water – Whole

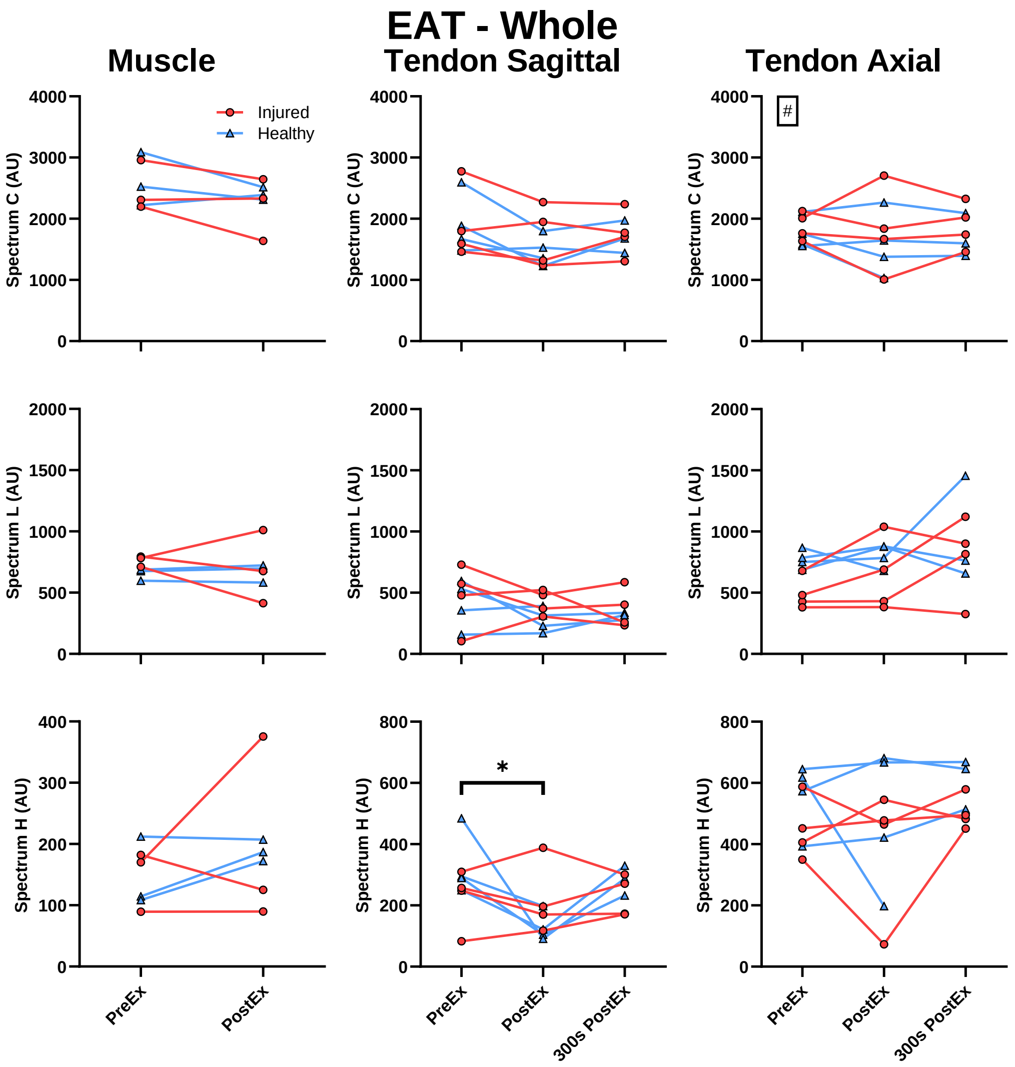

Figure S8. Individual datapoints for collagen (spectrum C), lipid (spectrum L) and water (spectrum H) related MSOT outcomes measured in the whole tissue region of the muscle and tendon in the EAT group, with the tendon measured both on sagittal and axial scans. * Significant effect of time. # Significant effect of side (injured/healthy). Significance markers placed in a box indicate a main effect not localized to a specific timepoint.

RPT

Blood – ROI

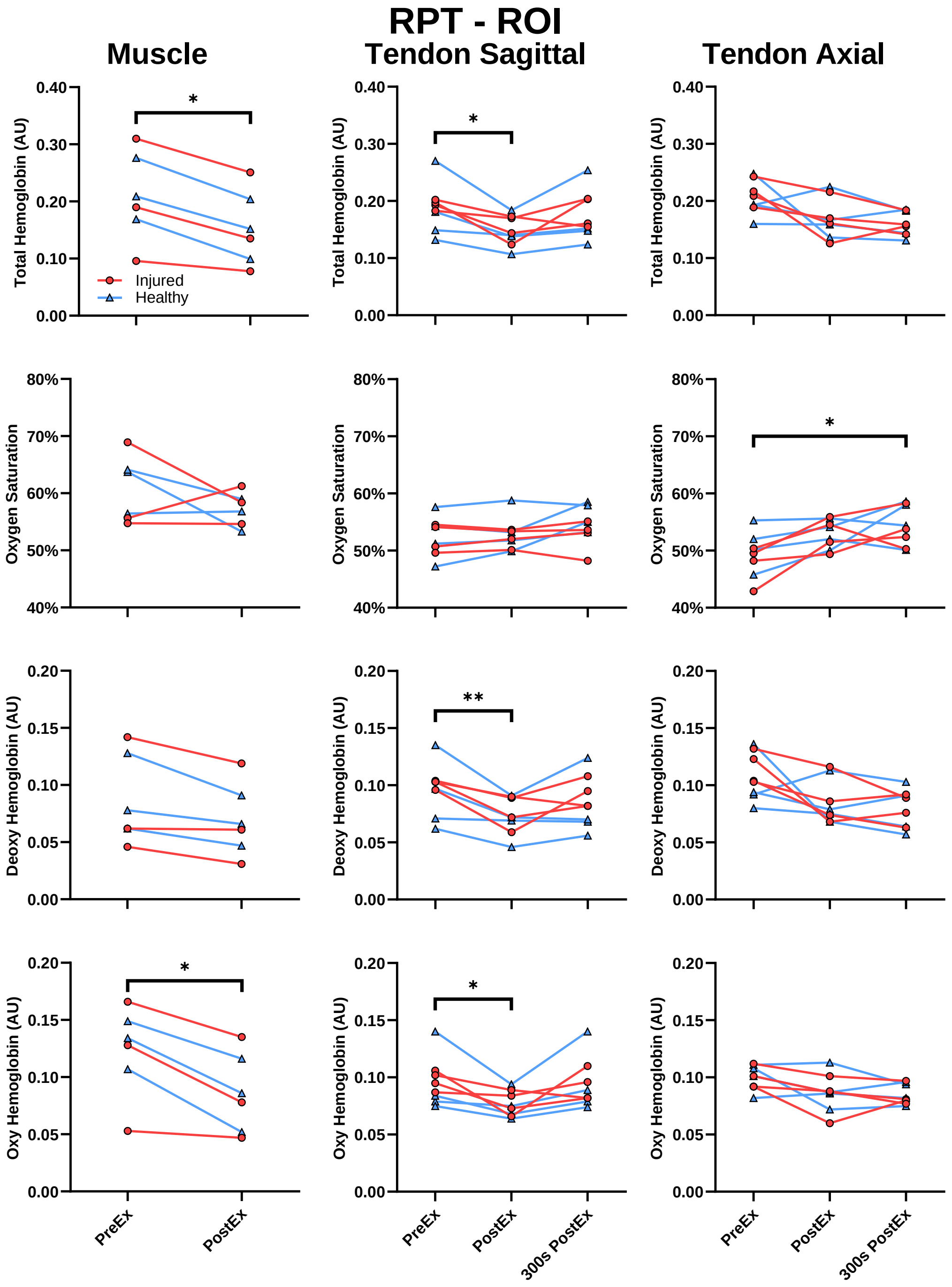

Figure S9. Individual datapoints for blood related MSOT outcomes measured in a smaller optimized ROI in the muscle and tendon of the RPT group, with the tendon measured both on sagittal and axial scans. * Significant effect of time.

Collagen, lipid, water – ROI

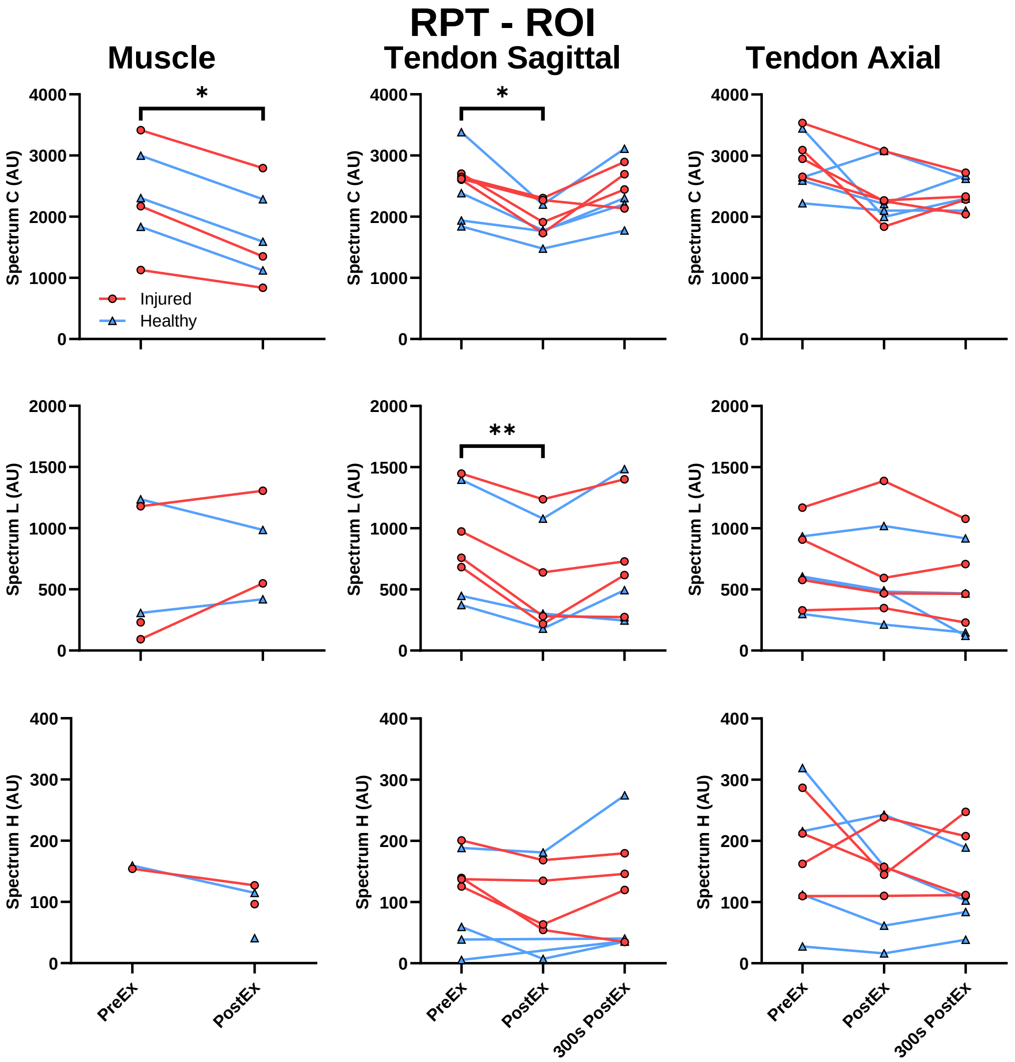

Figure S10. Individual datapoints for collagen (spectrum C), lipid (spectrum L) and water (spectrum H) related MSOT outcomes measured in a smaller optimized ROI in the muscle and tendon of the RPT group, with the tendon measured both on sagittal and axial scans. * Significant effect of time.

Blood – Whole

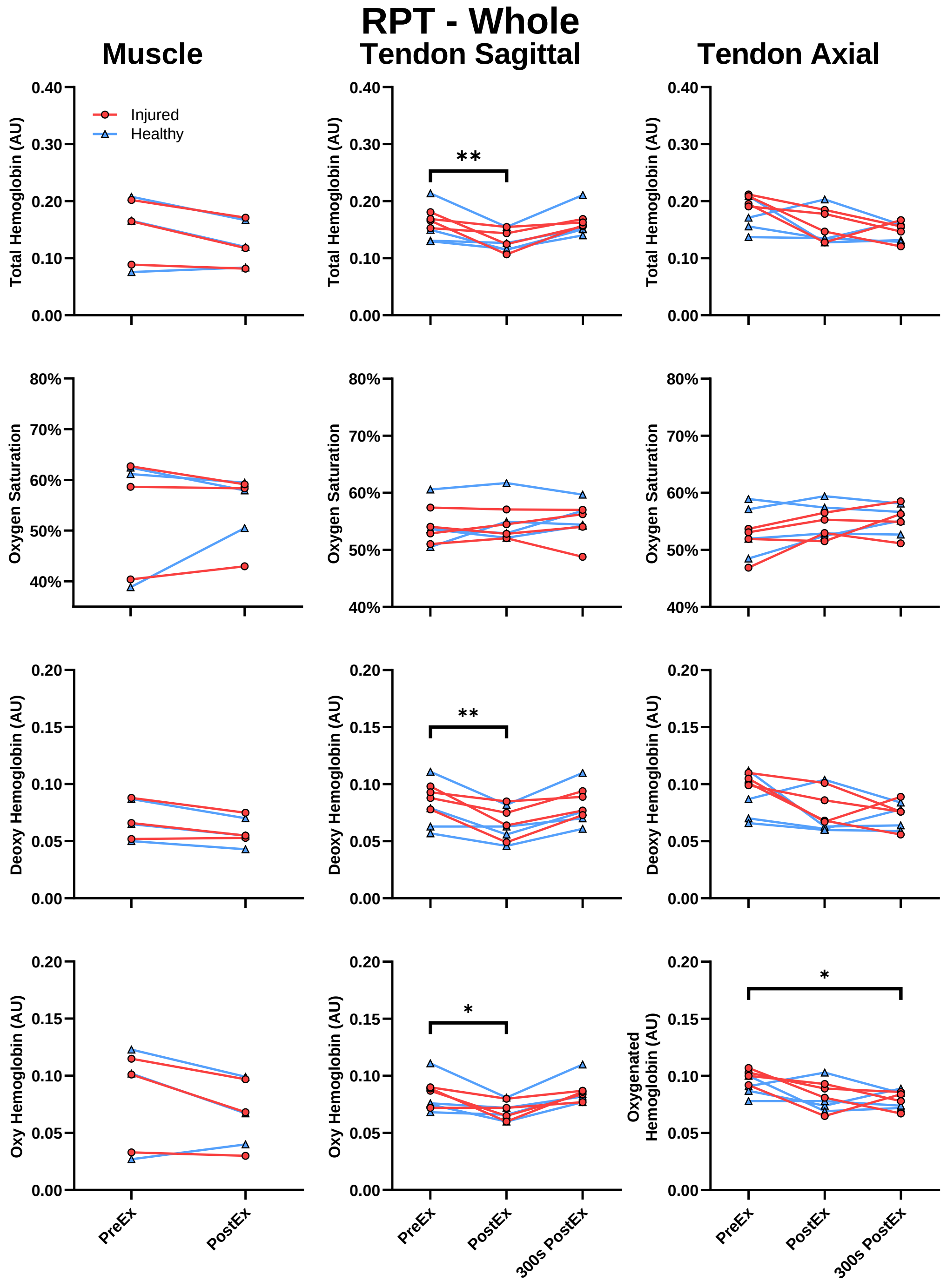

Figure S11. Individual datapoints for blood related MSOT outcomes measured in the whole tissue region of the muscle and tendon in the RPT group, with the tendon measured both on sagittal and axial scans. * Significant effect of time.

Collagen, lipid, water – Whole

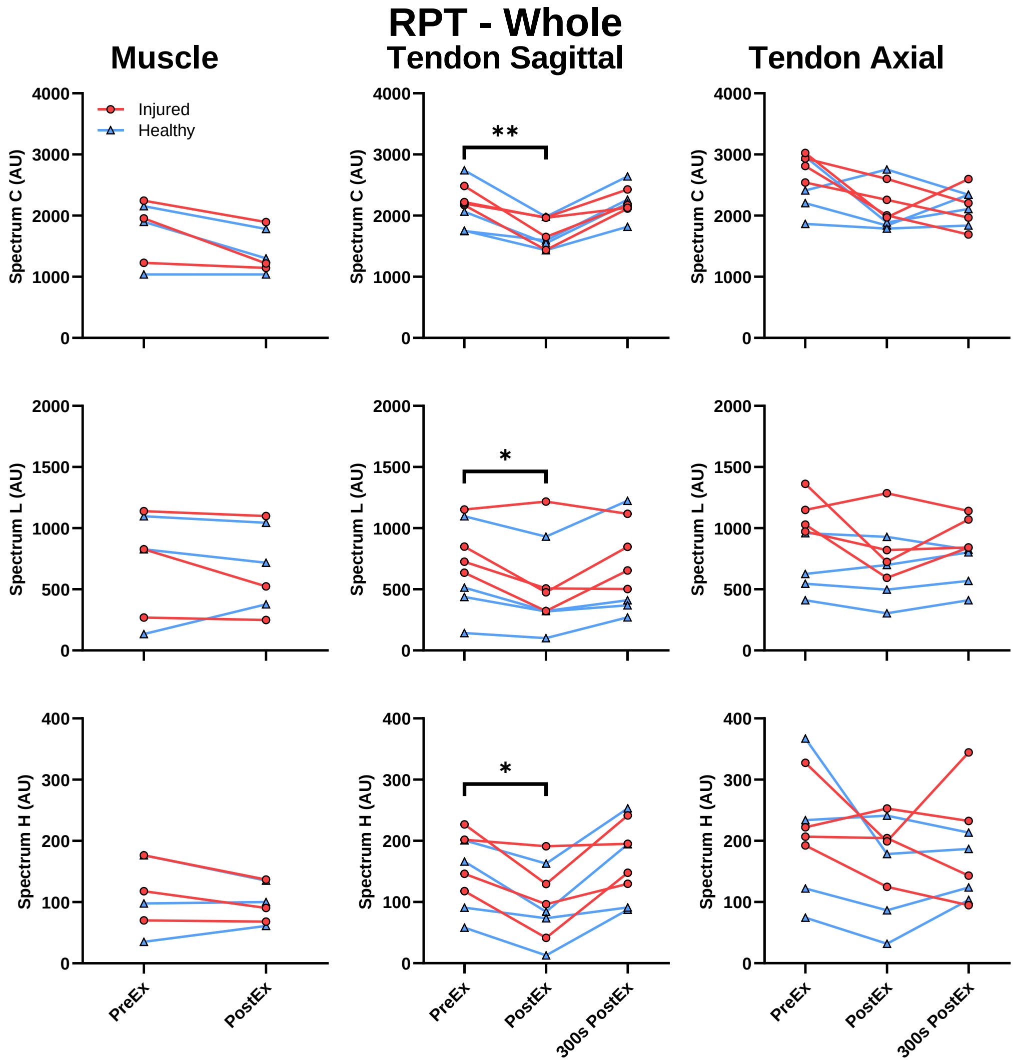

Figure S12. Individual datapoints for collagen (spectrum C), lipid (spectrum L) and water (spectrum H) related MSOT outcomes measured in the whole tissue region of the muscle and tendon in the RPT group, with the tendon measured both on sagittal and axial scans. * Significant effect of time.

ATR

Blood – ROI and Whole

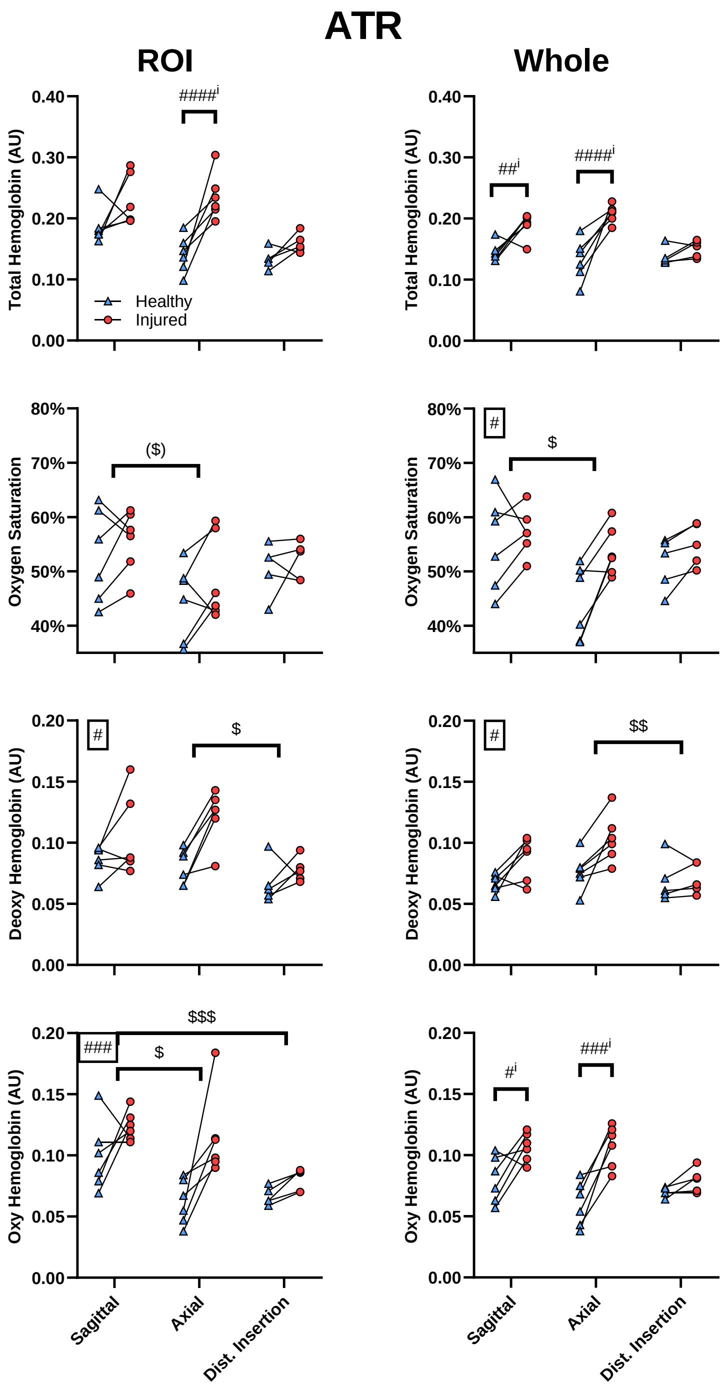

Figure S13. Individual datapoints for blood related MSOT outcomes measured in a smaller optimized ROI or the whole tissue region of the Achilles tendon rupture site in axial and sagittal scans and the distal insertion (uninjured site) for the ATR group. $ Significant effect of location (sagittal, axial, distal). # Significant effect of side (injured/healthy). [i] Significant interaction between location and side. (#)/($) 0.05<p<0.1.

Collagen, lipid and water – ROI and Whole

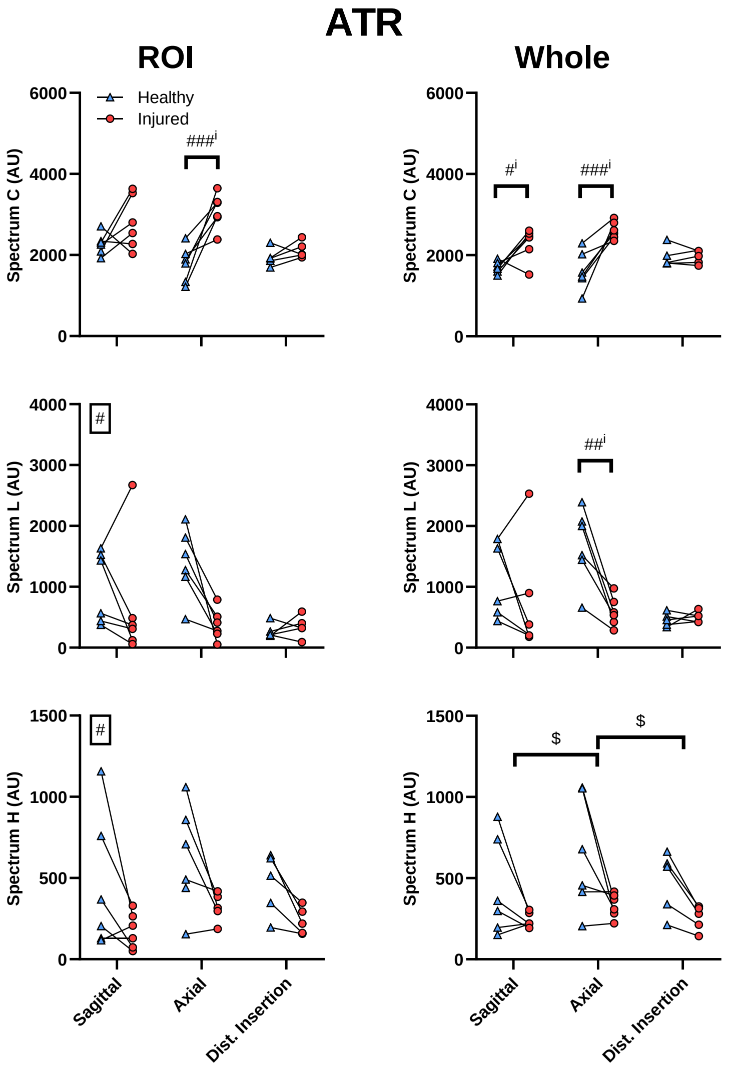

Figure S14. Individual datapoints for collagen (spectrum C), lipid (spectrum L) and water (spectrum H) related MSOT outcomes measured in a smaller optimized ROI or the whole tissue region of the Achilles tendon rupture site in axial and sagittal scans and the distal insertion (uninjured site) for the ATR group. # Significant effect of side (injured/healthy). $ Significant effect of location (sagittal, axial, distal). [i] Significant interaction between location and side.

MSI

Blood – ROI

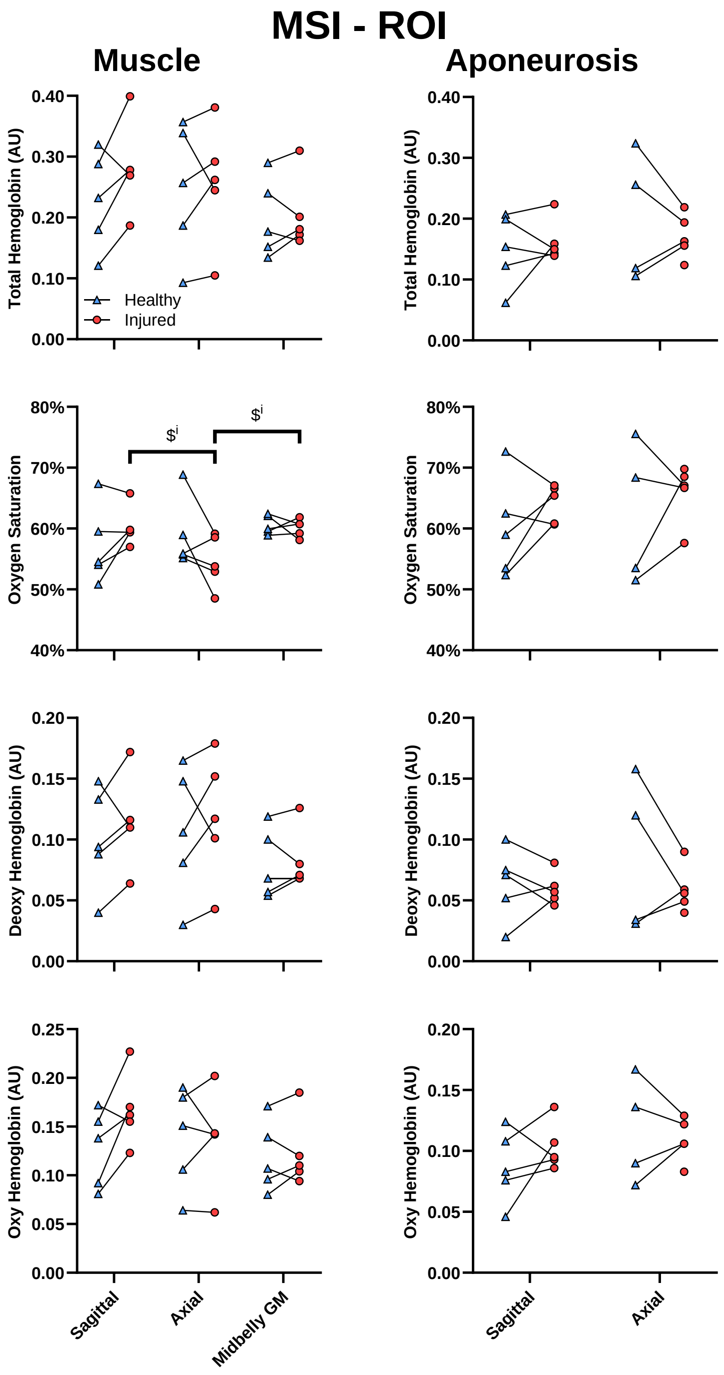

Figure S15. Individual datapoints for blood related MSOT outcomes measured in a smaller optimized ROI in the aponeurosis and distal GM muscle (injury site) for both axial and sagittal scans and the midbelly GM muscle (uninjured site) in the MSI group. $ Significant effect of location (sagittal, axial, midbelly). [i] Significant interaction between location and side.

Collagen, lipid, water – ROI

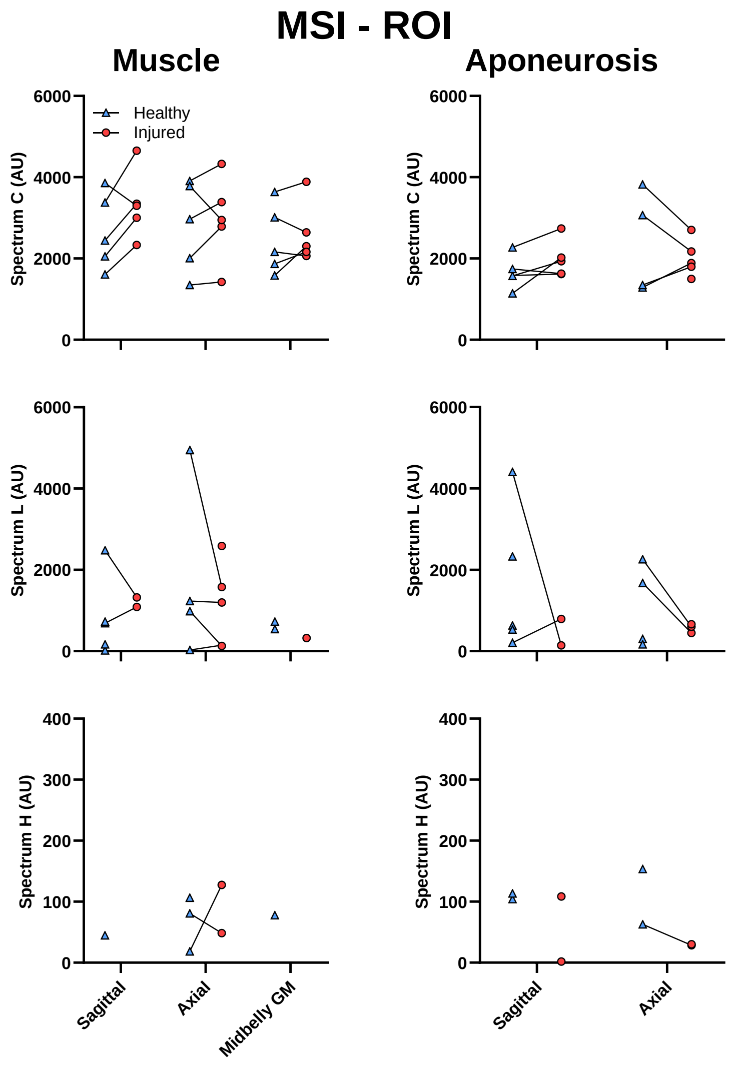

Figure S16. Individual datapoints for collagen (spectrum C), lipid (spectrum L) and water (spectrum H) related MSOT outcomes measured in a smaller optimized ROI in the aponeurosis and distal GM muscle (injury site) for both axial and sagittal scans and the midbelly GM muscle (uninjured site) in the MSI group.

Blood – Whole

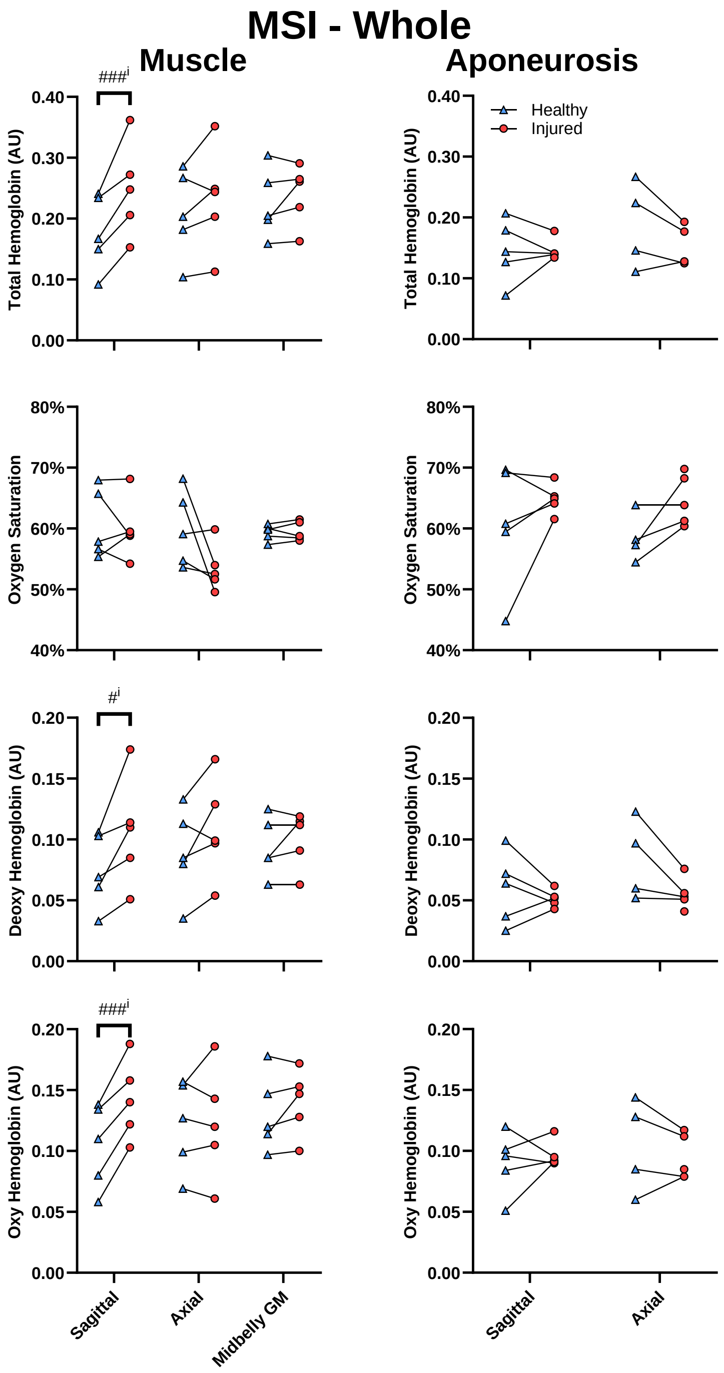

Figure S17. Individual datapoints for blood related MSOT outcomes measured in the whole tissue region of the aponeurosis and distal GM muscle (injury site) for both axial and sagittal scans and the midbelly GM muscle (uninjured site) in the MSI group. # Significant effect of side (injured/healthy). [i] Significant interaction between location and side.

Collagen, lipid, water – Whole

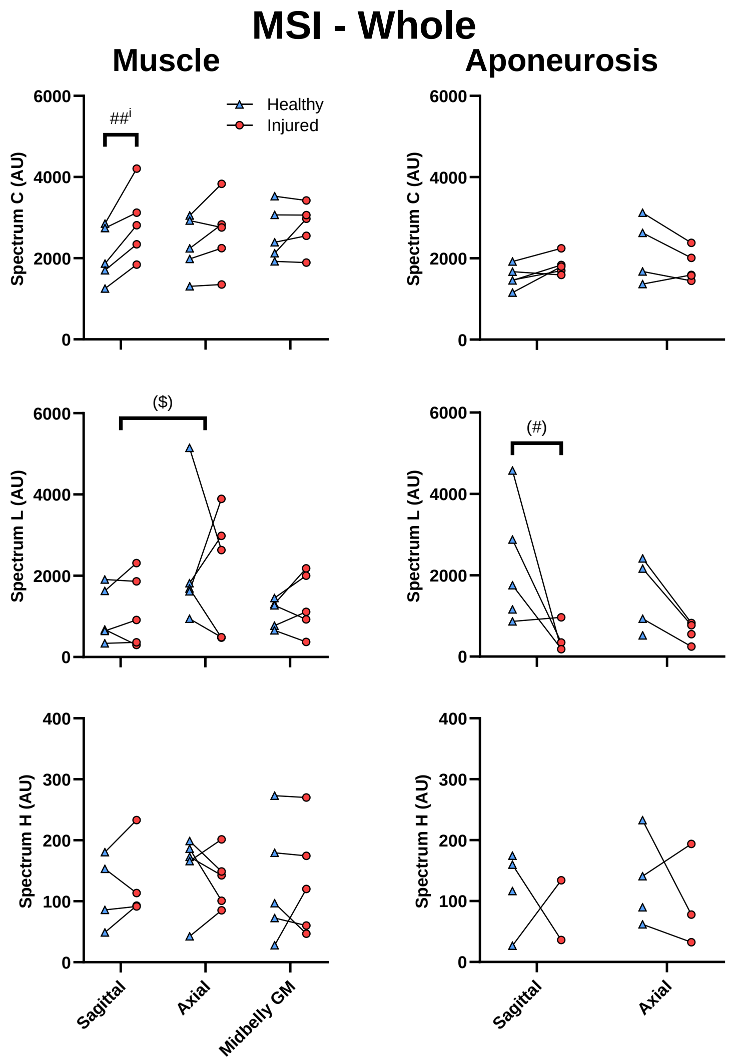

Figure S18. Individual datapoints for collagen (spectrum C), lipid (spectrum L) and water (spectrum H) related MSOT outcomes measured in the whole tissue region of the aponeurosis and distal GM muscle (injury site) for both axial and sagittal scans and the midbelly GM muscle (uninjured site) in the MSI group. $ Significant effect of location (sagittal, axial, midbelly). # Significant effect of side (injured/healthy). [i] Significant interaction between location and side. (#)/($) 0.05<p<0.1.

MSOT image examples

CPT – Injured – Collagen, lipid – Sagittal

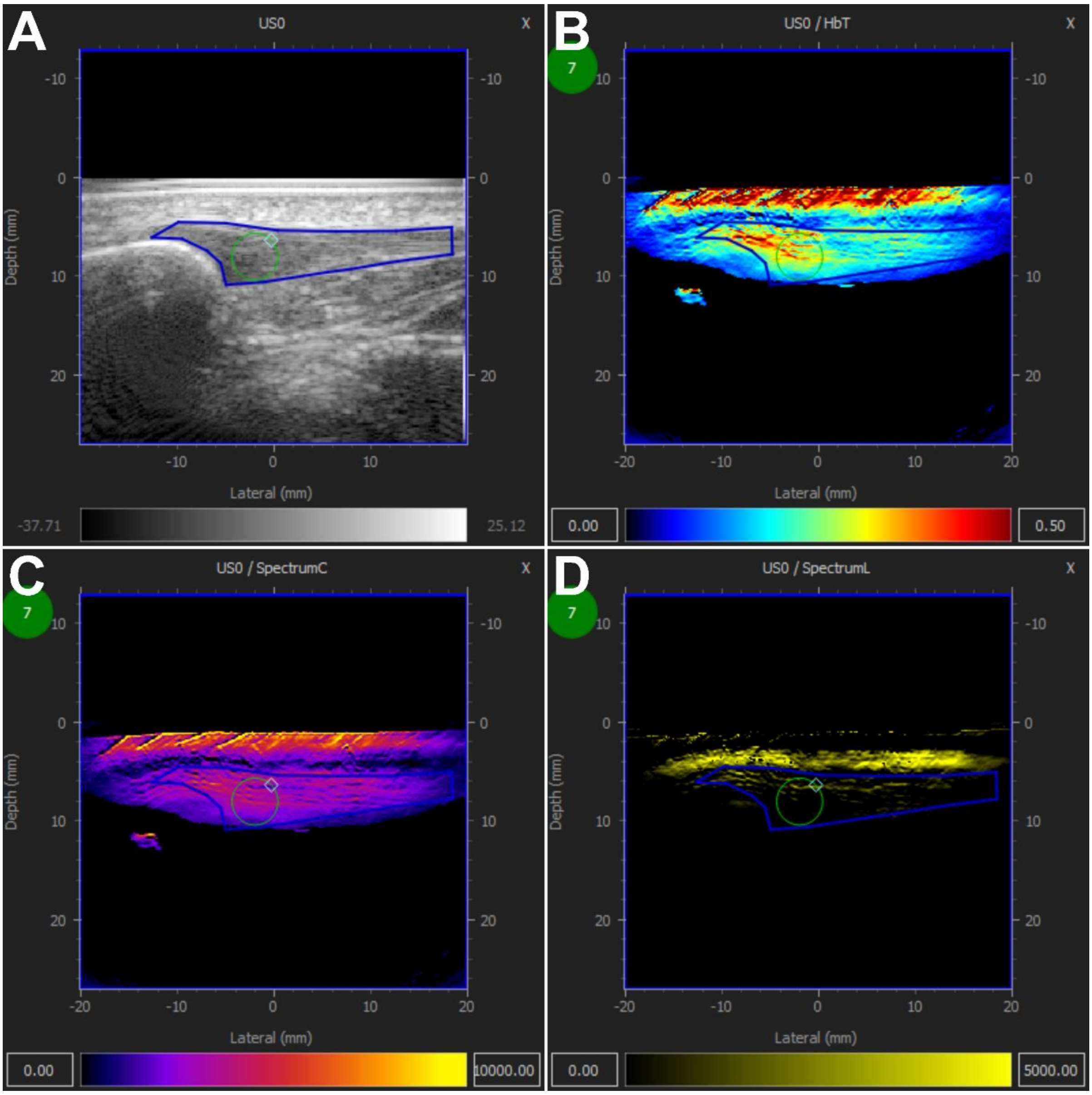

Figure S19. Example of a sagittal MSOT image on the patellar tendon of a patient with chronic patellar tendinopathy (CPT). A) B-mode. B) Total hemoglobin. C) Collagen (Spectrum C). D) Lipid (Spectrum L). ROIs delineating the entire visible tendon (blue) and a smaller central region (green) are shown.

CPT – Injured – Blood – Axial

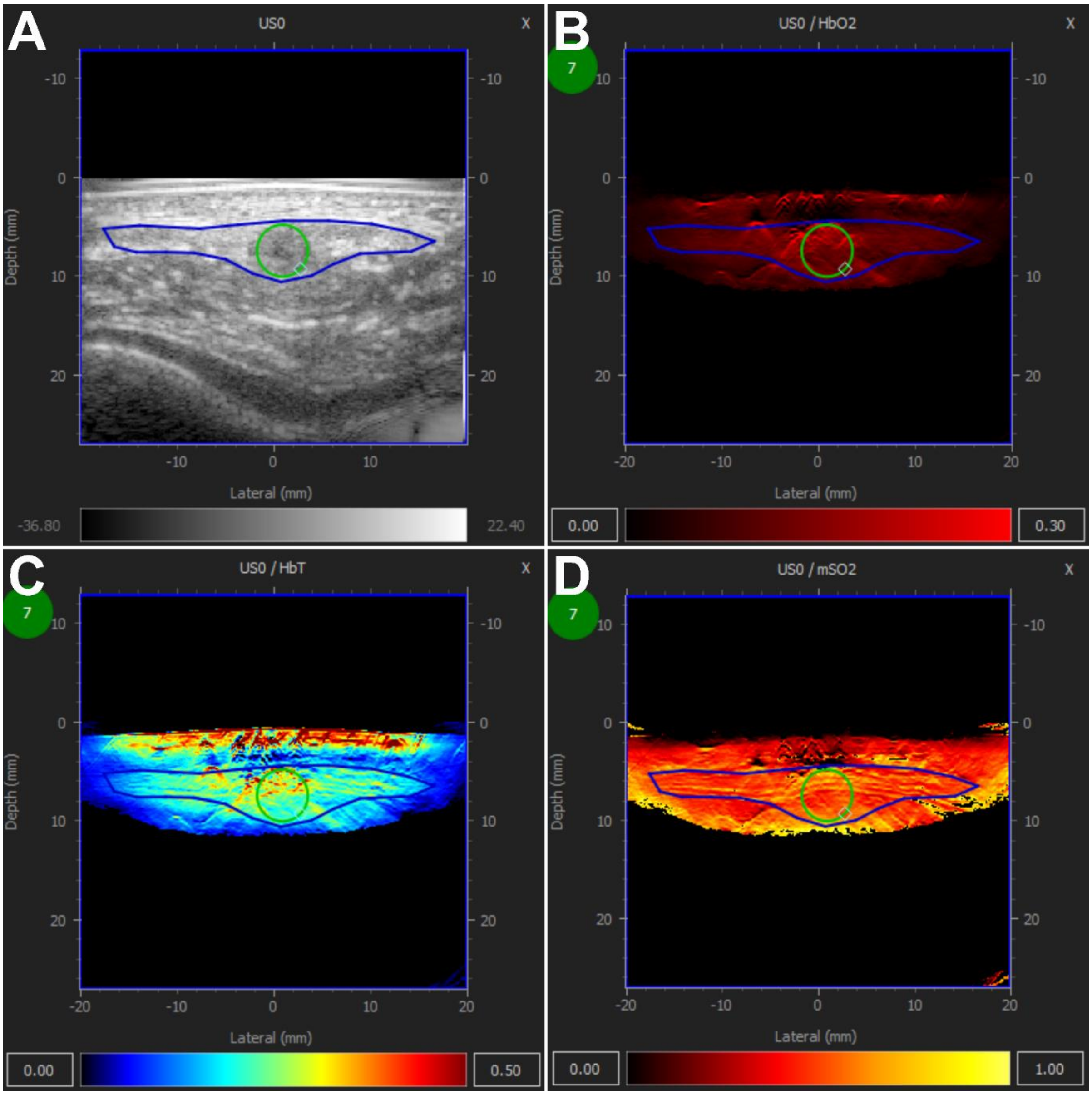

Figure S20. Example of an axial MSOT image on the patellar tendon of a patient with chronic patellar tendinopathy (CPT). A) B-mode. B) Oxygenated hemoglobin. C) Total hemoglobin. D) Oxygen saturation. ROIs delineating the entire visible tendon (blue) and a smaller central region (green) are shown.

CPT – Injured – Collagen, lipid – Axial

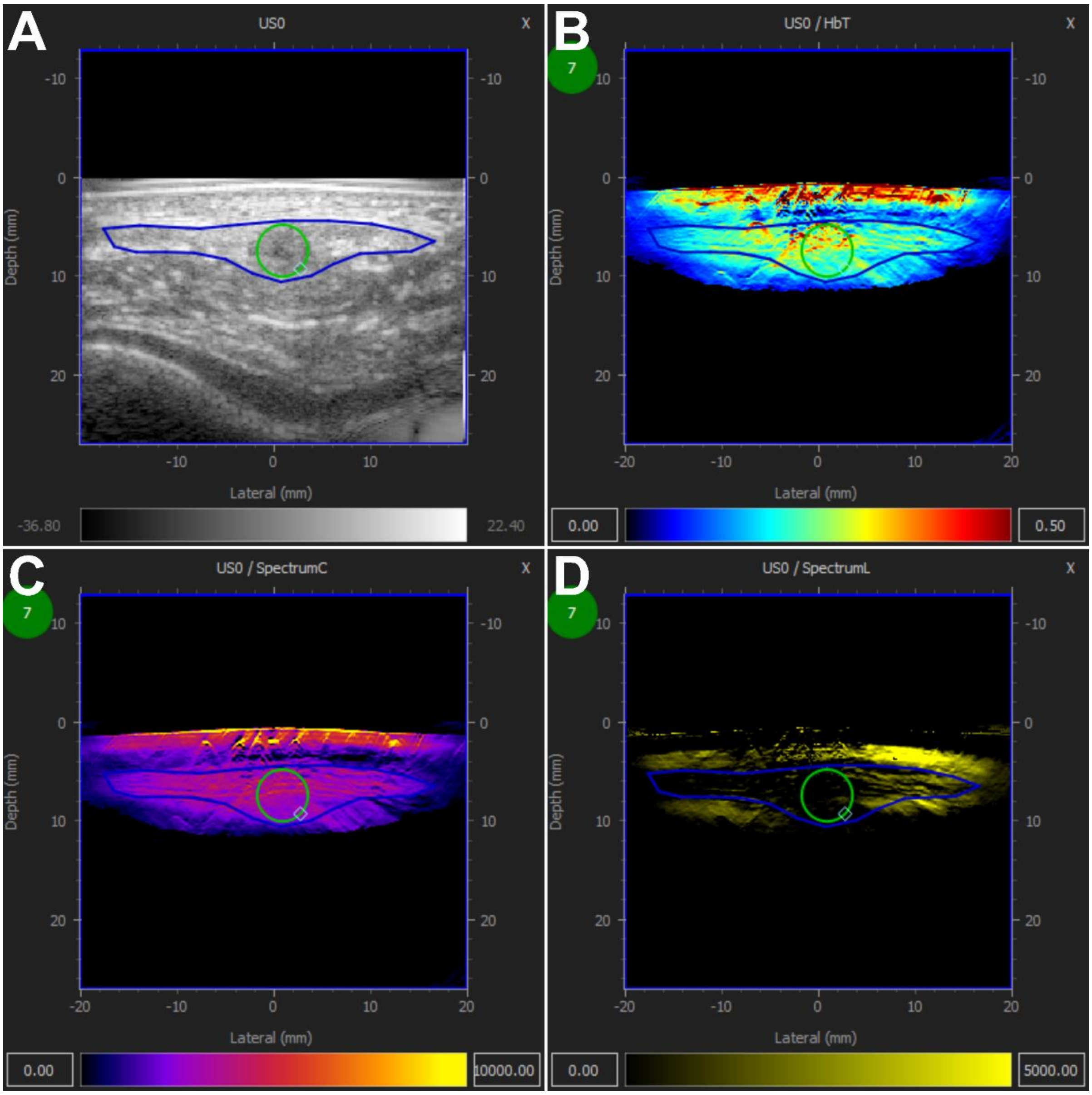

Figure S21. Example of an axial MSOT image on the patellar tendon of a patient with chronic patellar tendinopathy (CPT). A) B-mode. B) Total hemoglobin. C) Collagen (Spectrum C). D) Lipid (Spectrum L). ROIs delineating the entire visible tendon (blue) and a smaller central region (green) are shown.

CPT – Healthy – Collagen, lipid – Sagittal

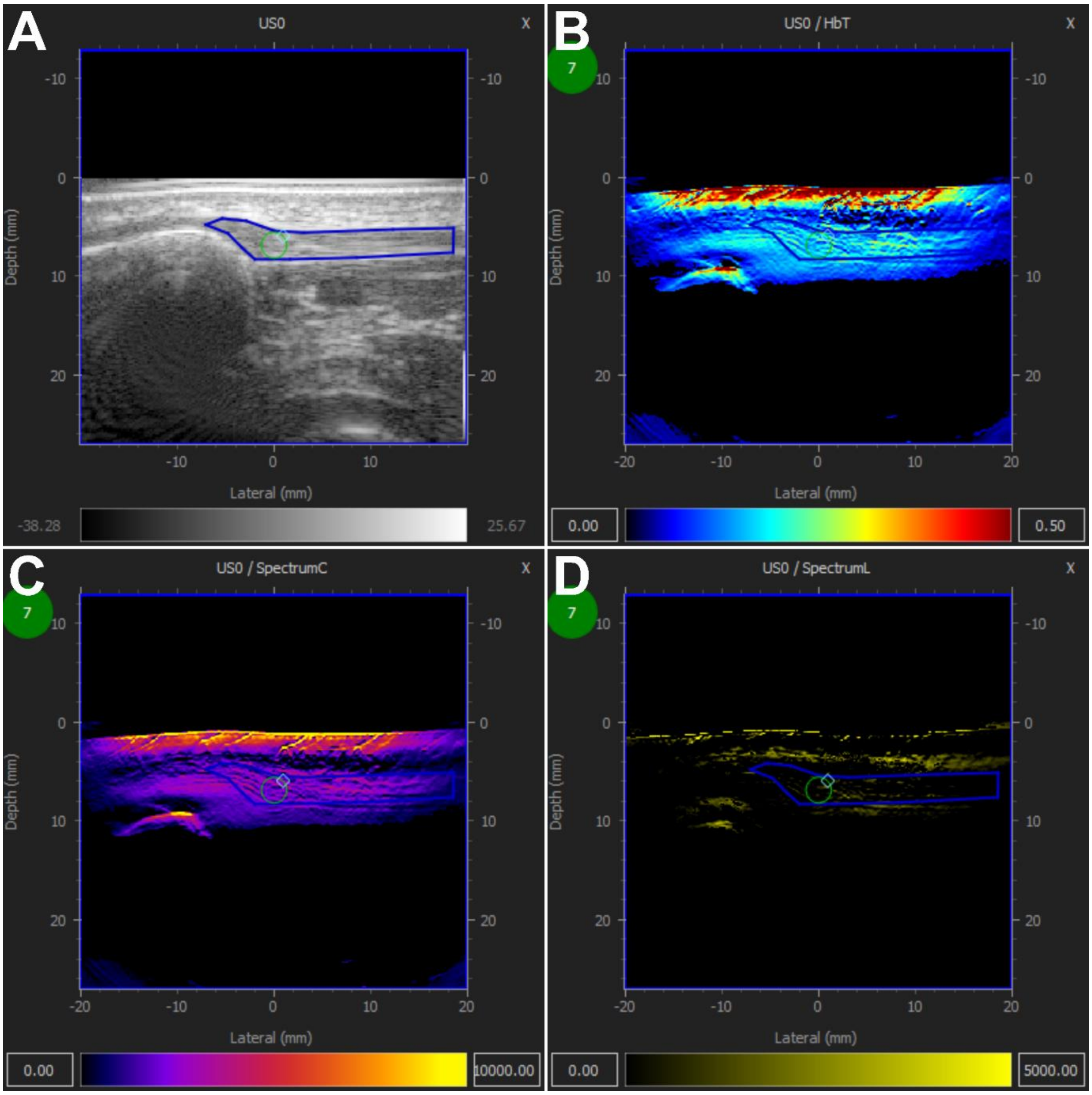

Figure S22. Example of a sagittal MSOT image on the uninjured contralateral patellar tendon of a chronic patellar tendinopathy patient (CPT). A) B-mode. B) Total hemoglobin. C) Collagen (Spectrum C). D) Lipid (Spectrum L). ROIs delineating the entire visible tendon (blue) and a smaller central region (green) are shown.

CPT – Healthy – Blood – Axial

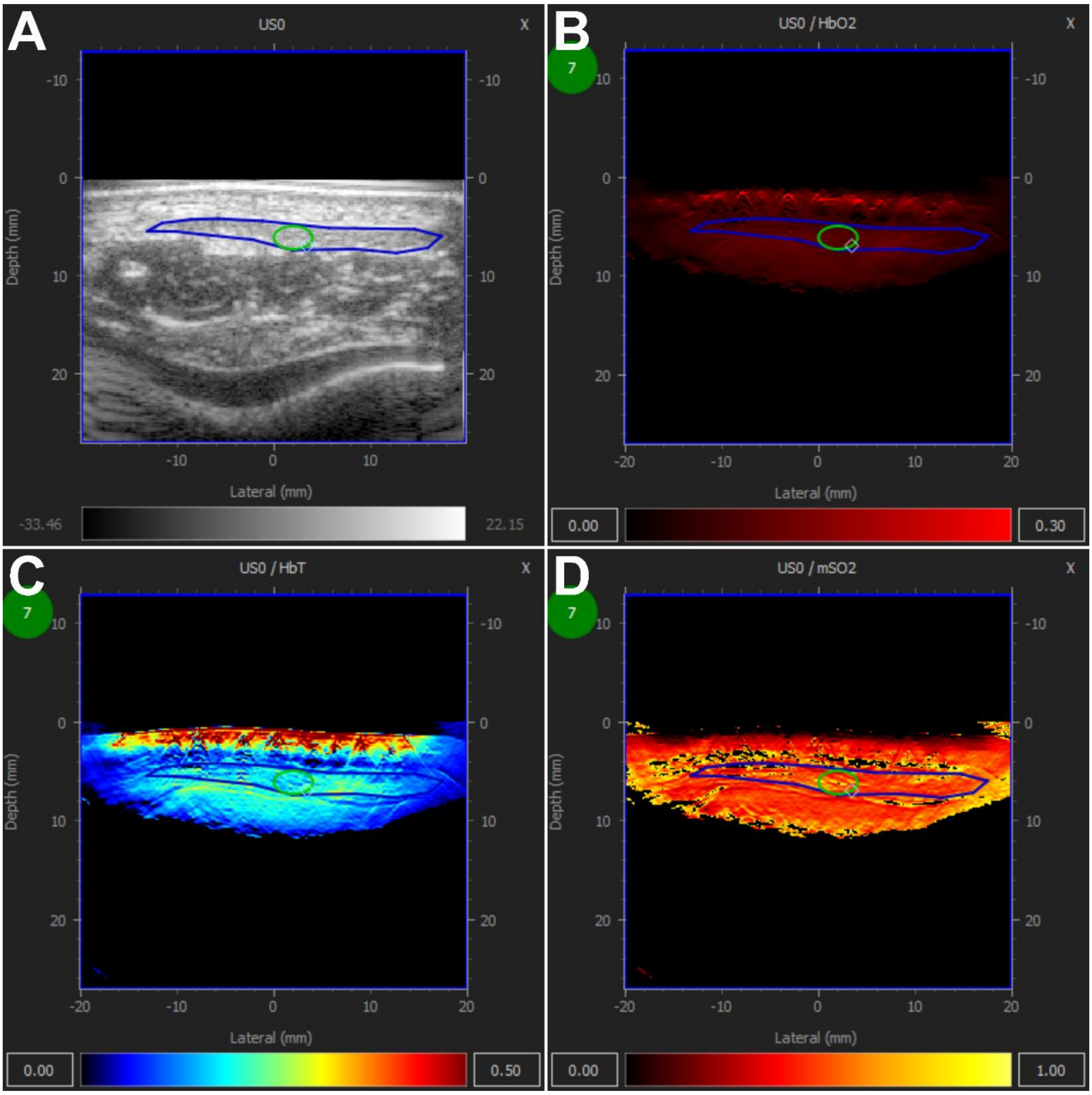

Figure S23. Example of an axial MSOT image on the uninjured contralateral patellar tendon of a chronic patellar tendinopathy patient (CPT). A) B-mode. B) Oxygenated hemoglobin. C) Total hemoglobin. D) Oxygen saturation. ROIs delineating the entire visible tendon (blue) and a smaller central region (green) are shown.

CPT – Healthy – Collagen, lipid – Axial

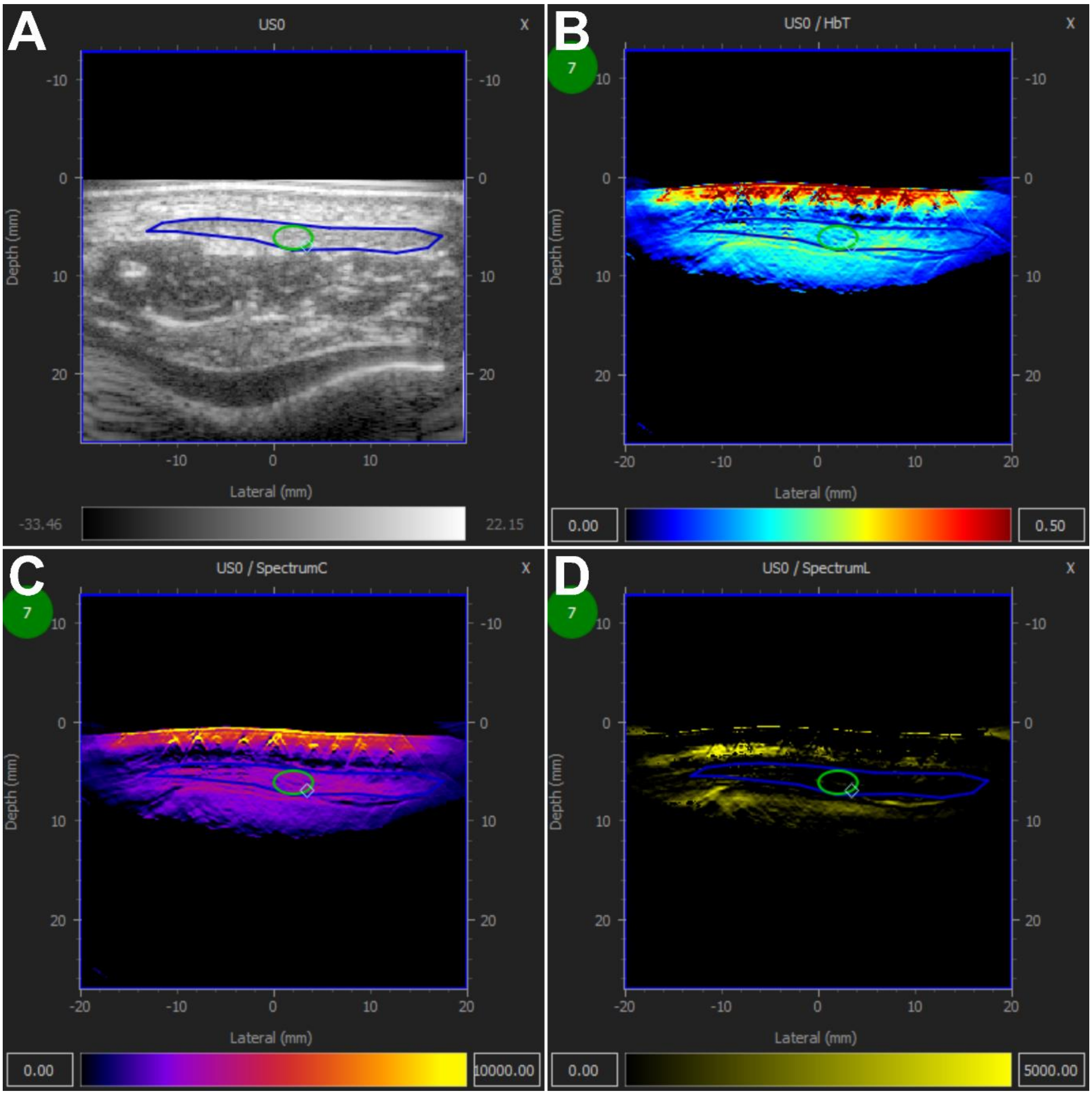

Figure S24. Example of an axial MSOT image on the uninjured contralateral patellar tendon of a chronic patellar tendinopathy patient (CPT). A) B-mode. B) Total hemoglobin. C) Collagen (Spectrum C). D) Lipid (Spectrum L). ROIs delineating the entire visible tendon (blue) and a smaller central region (green) are shown.

ATR – Inured – Blood – Sagittal

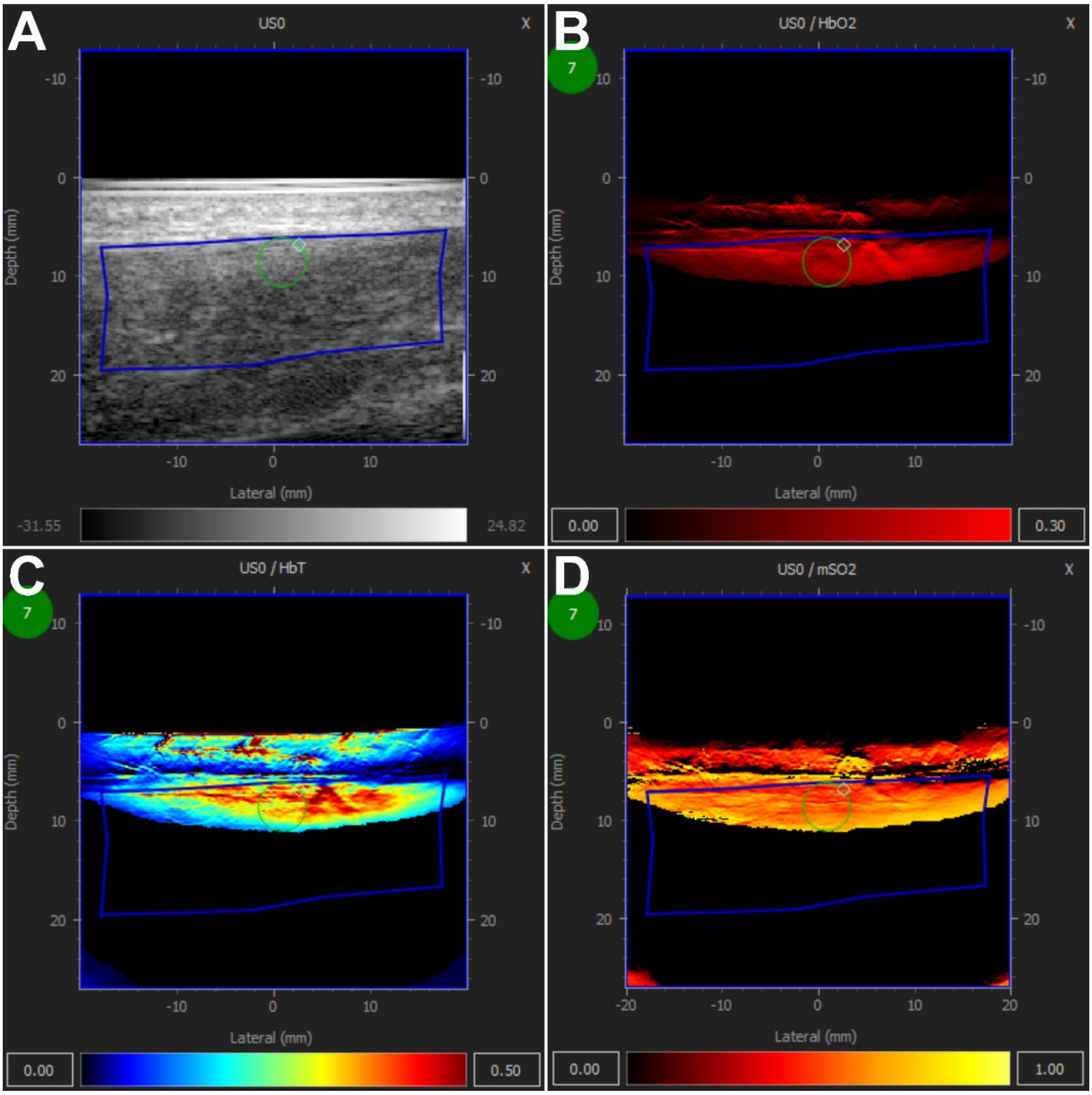

Figure S25. Example of a sagittal MSOT image on the injured Achilles tendon of an Achilles tendon rupture patient (ATR). A) B-mode. B) Oxygenated hemoglobin. C) Total hemoglobin. D) Oxygen saturation. ROIs delineating the entire visible tendon (blue) and a smaller central region (green) are shown.

ATR – Injured – Collagen, lipid – Axial

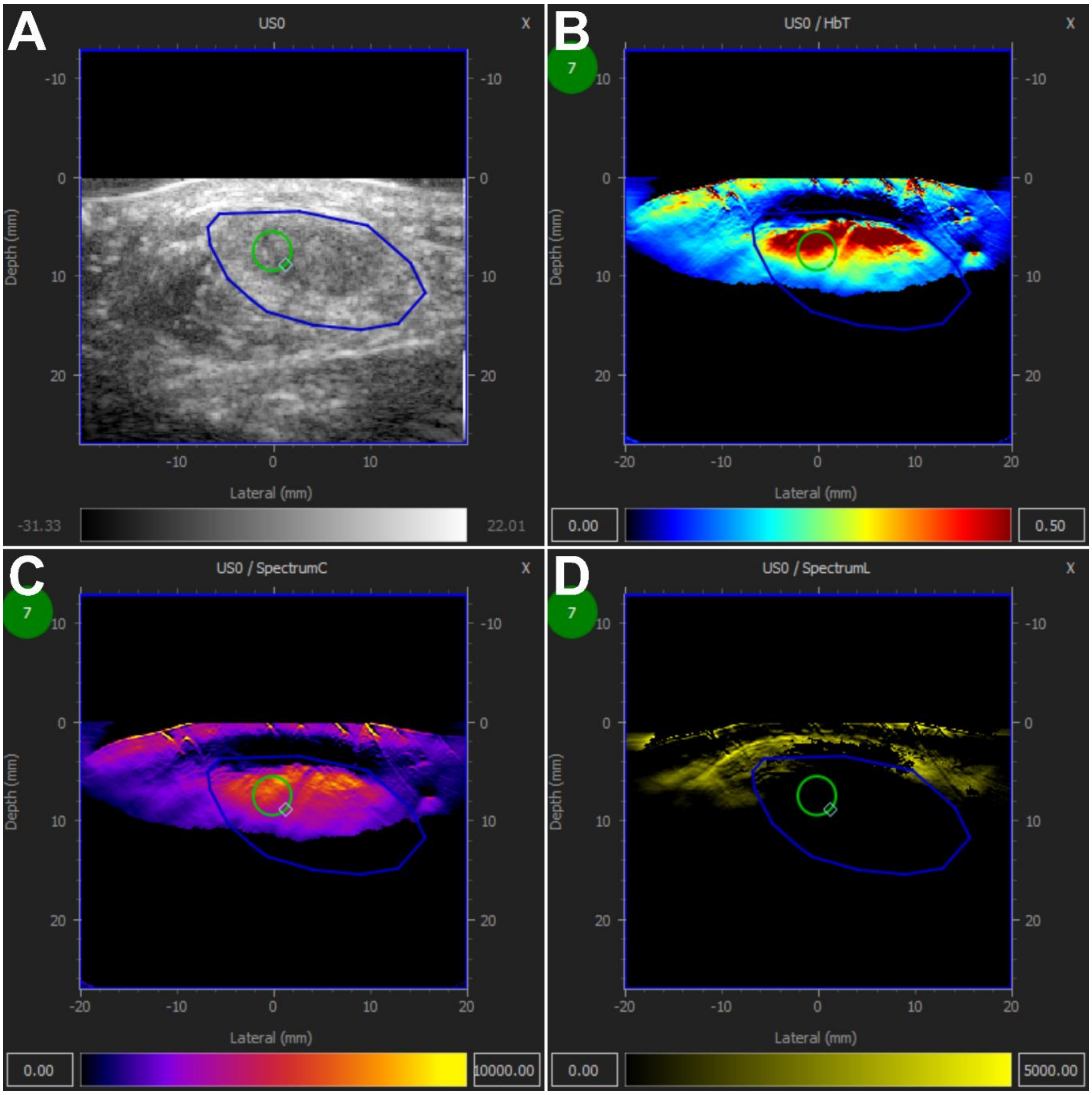

Figure S26. Example of an axial MSOT image on the injured Achilles tendon of an Achilles tendon rupture patient (ATR). A) B-mode. B) Total hemoglobin. C) Collagen (Spectrum C). D) Lipid (Spectrum L). ROIs delineating the entire visible tendon (blue) and a smaller central region (green) are shown.

ATR – Injured – Blood – Axial

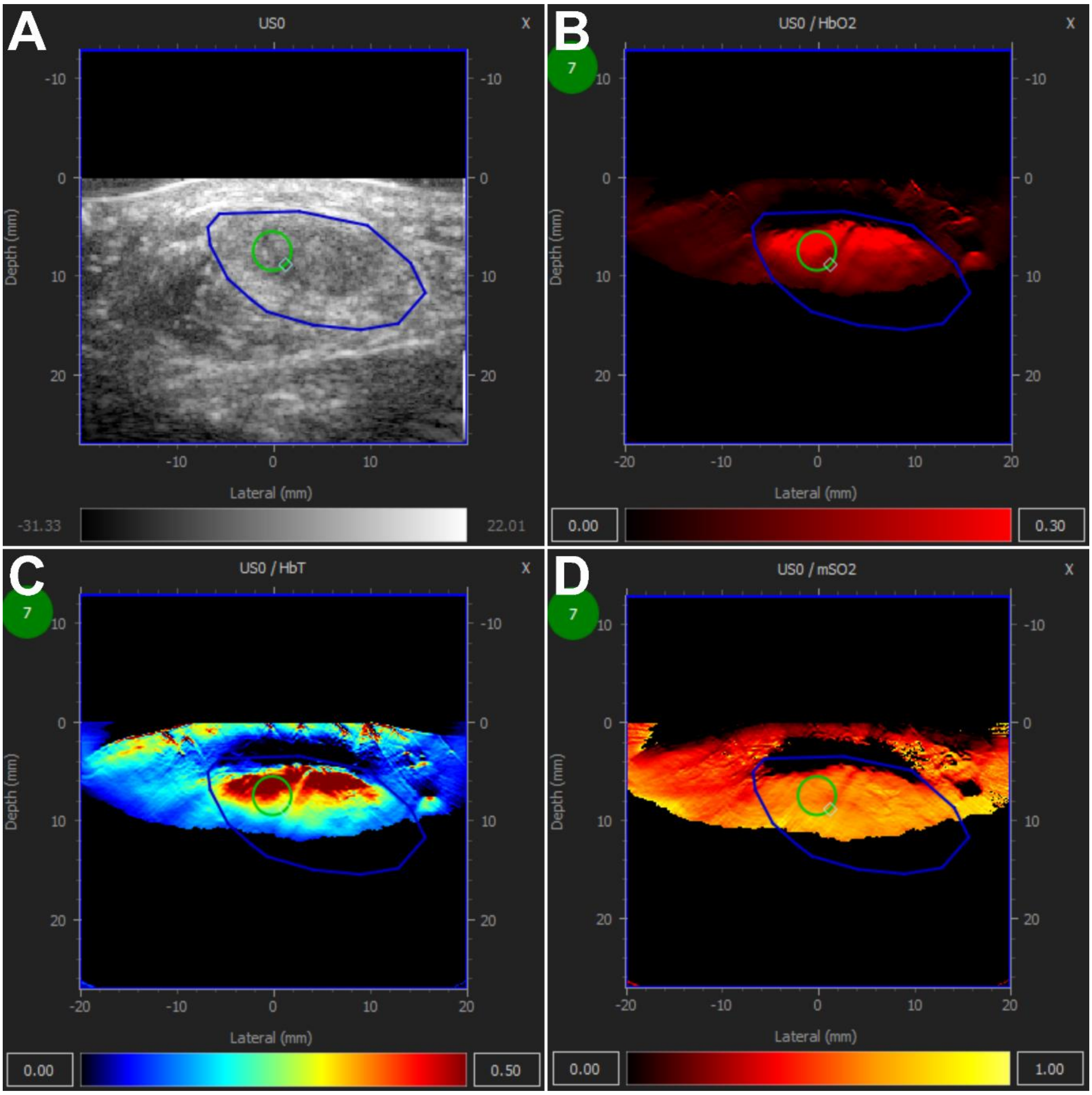

Figure S27. Example of an axial MSOT image on the injured Achilles tendon of an Achilles tendon rupture patient (ATR). A) B-mode. B) Oxygenated hemoglobin. C) Total hemoglobin. D) Oxygen saturation. ROIs delineating the entire visible tendon (blue) and a smaller central region (green) are shown.

ATR – Healthy – Blood – Sagittal

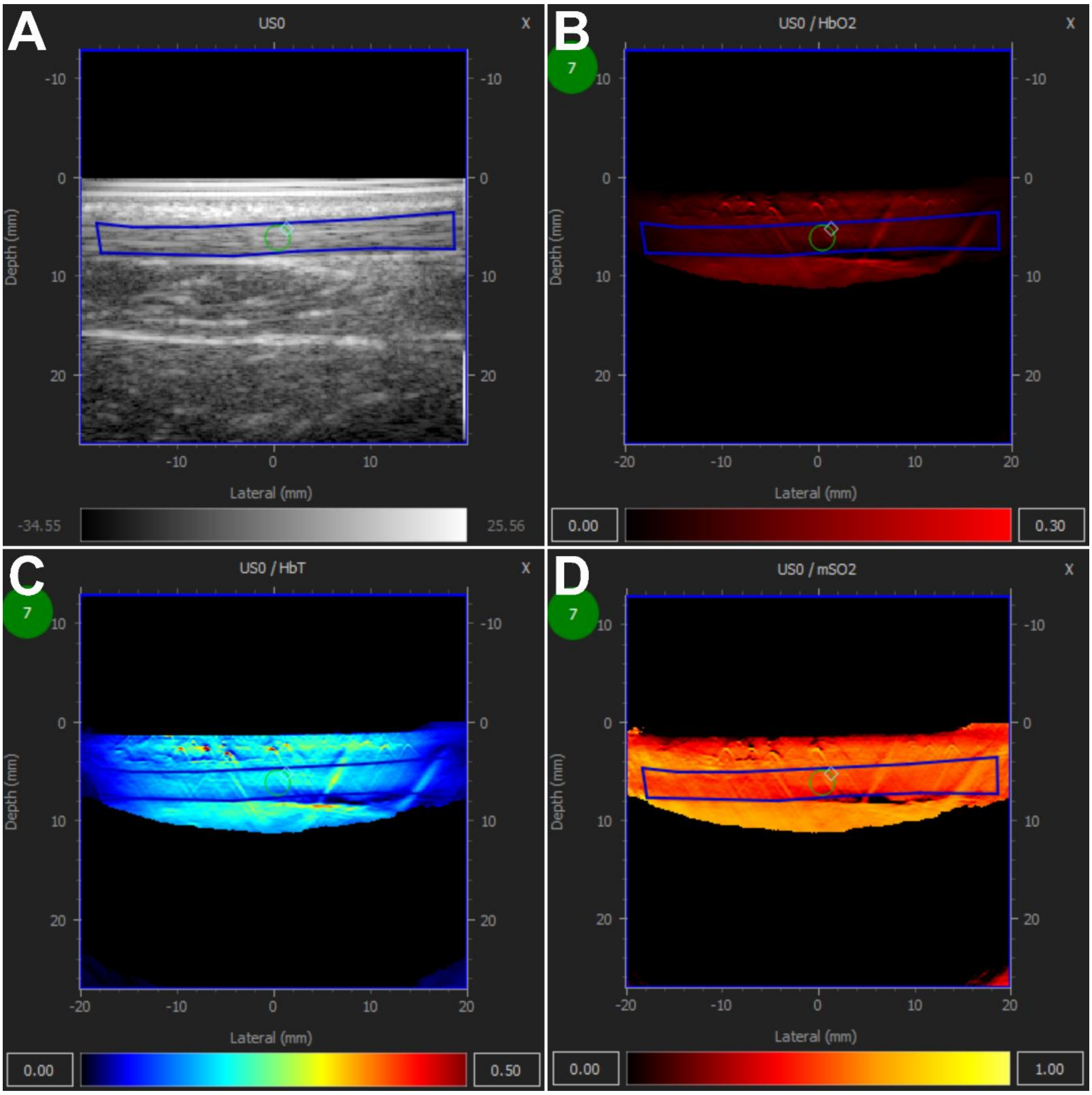

Figure S28. Example of a sagittal MSOT image on the healthy contralateral Achilles tendon of an Achilles tendon rupture patient (ATR). A) B-mode. B) Oxygenated hemoglobin. C) Total hemoglobin. D) Oxygen saturation. ROIs delineating the entire visible tendon (blue) and a smaller central region (green) are shown.

ATR – Healthy – Collagen, lipid – Axial

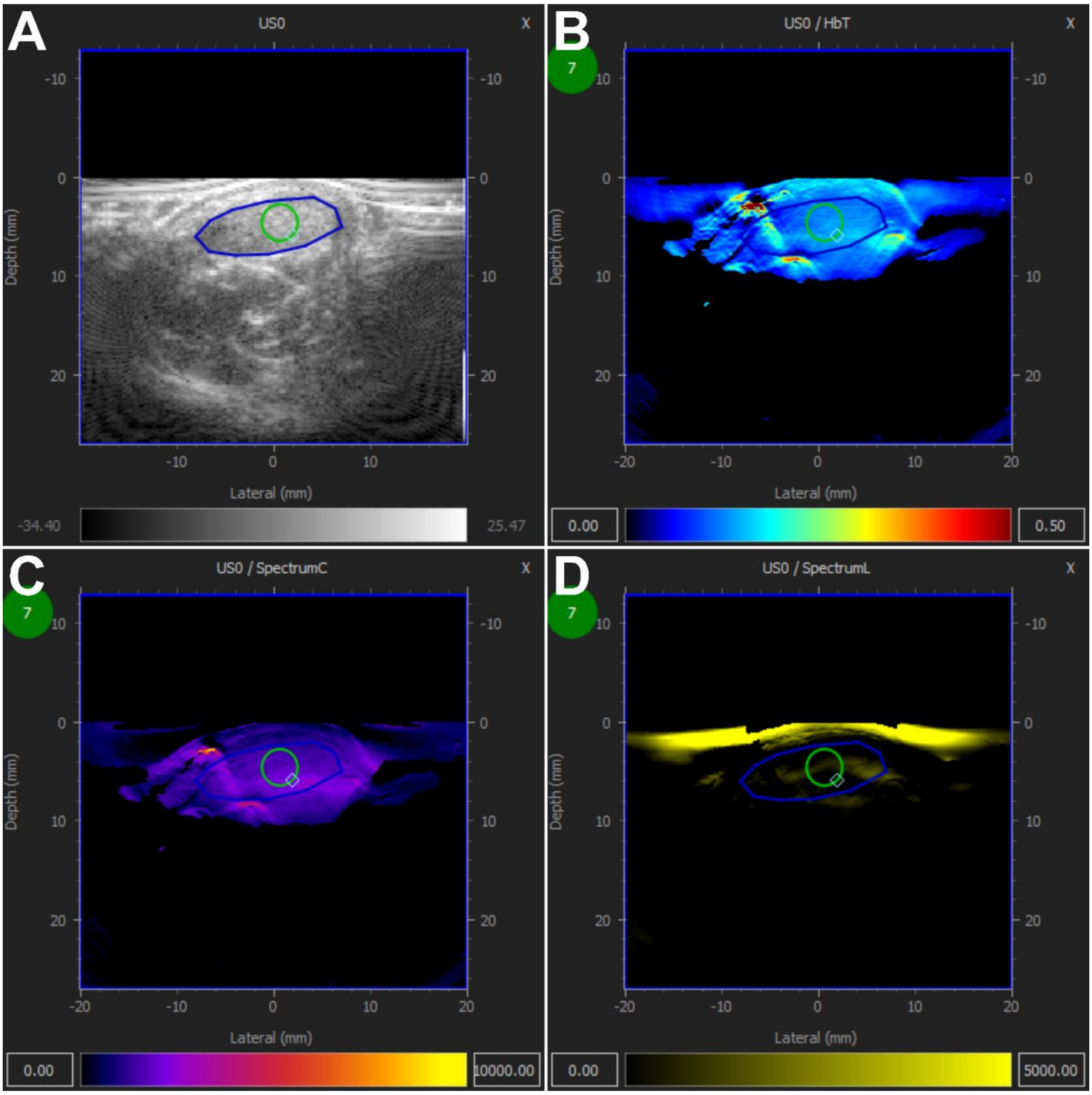

Figure S29. Example of an axial MSOT image on the healthy contralateral Achilles tendon of an Achilles tendon rupture patient (ATR). A) B-mode. B) Total hemoglobin. C) Collagen (Spectrum C). D) Lipid (Spectrum L). ROIs delineating the entire visible tendon (blue) and a smaller central region (green) are shown.

ATR – Healthy – Blood – Axial

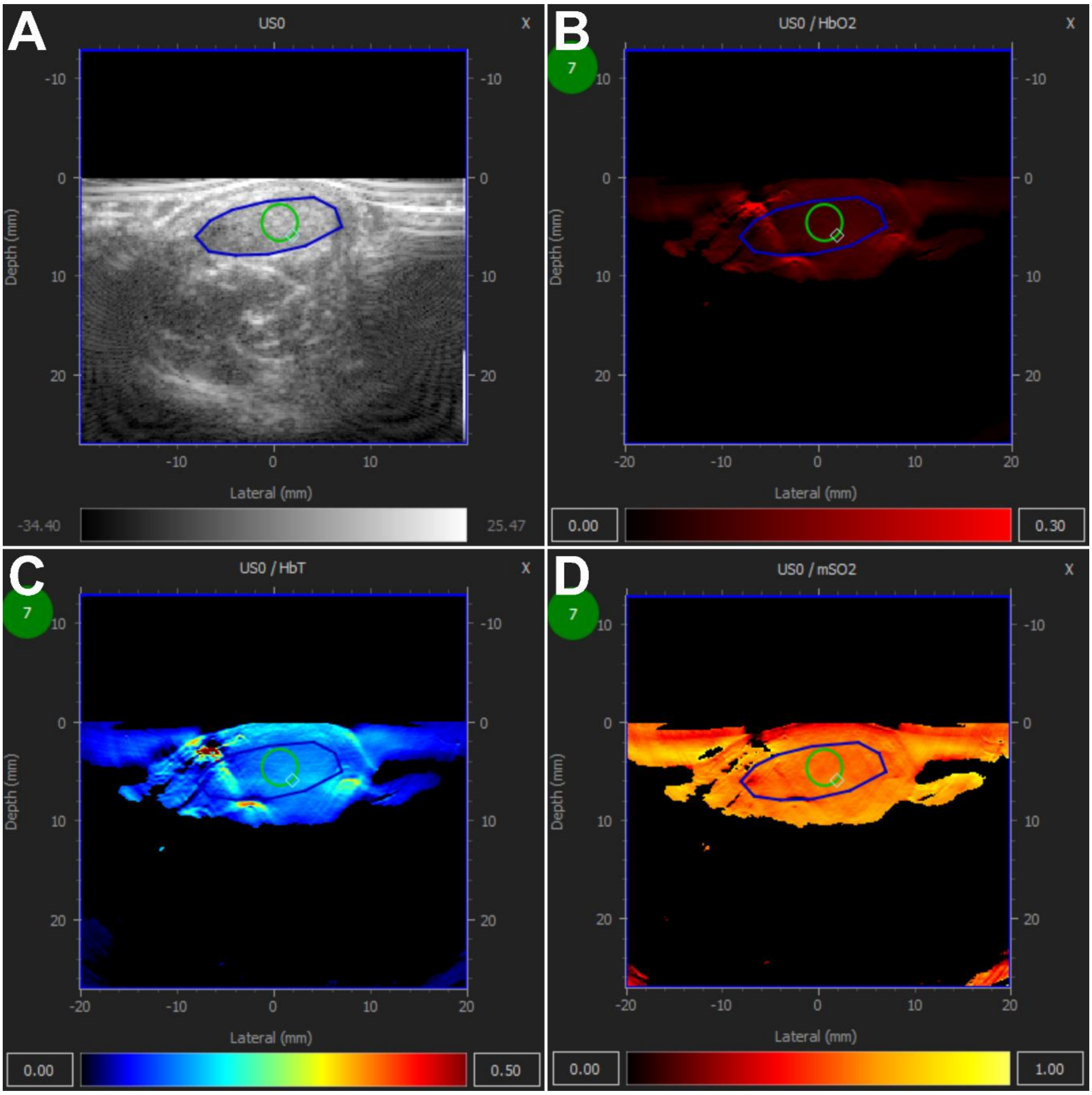

Figure S30. Example of an axial MSOT image on the healthy contralateral Achilles tendon of an Achilles tendon rupture patient (ATR). A) B-mode. B) Oxygenated hemoglobin. C) Total hemoglobin. D) Oxygen saturation. ROIs delineating the entire visible tendon (blue) and a smaller central region (green) are shown.

MSI – Inured – Blood – Sagittal

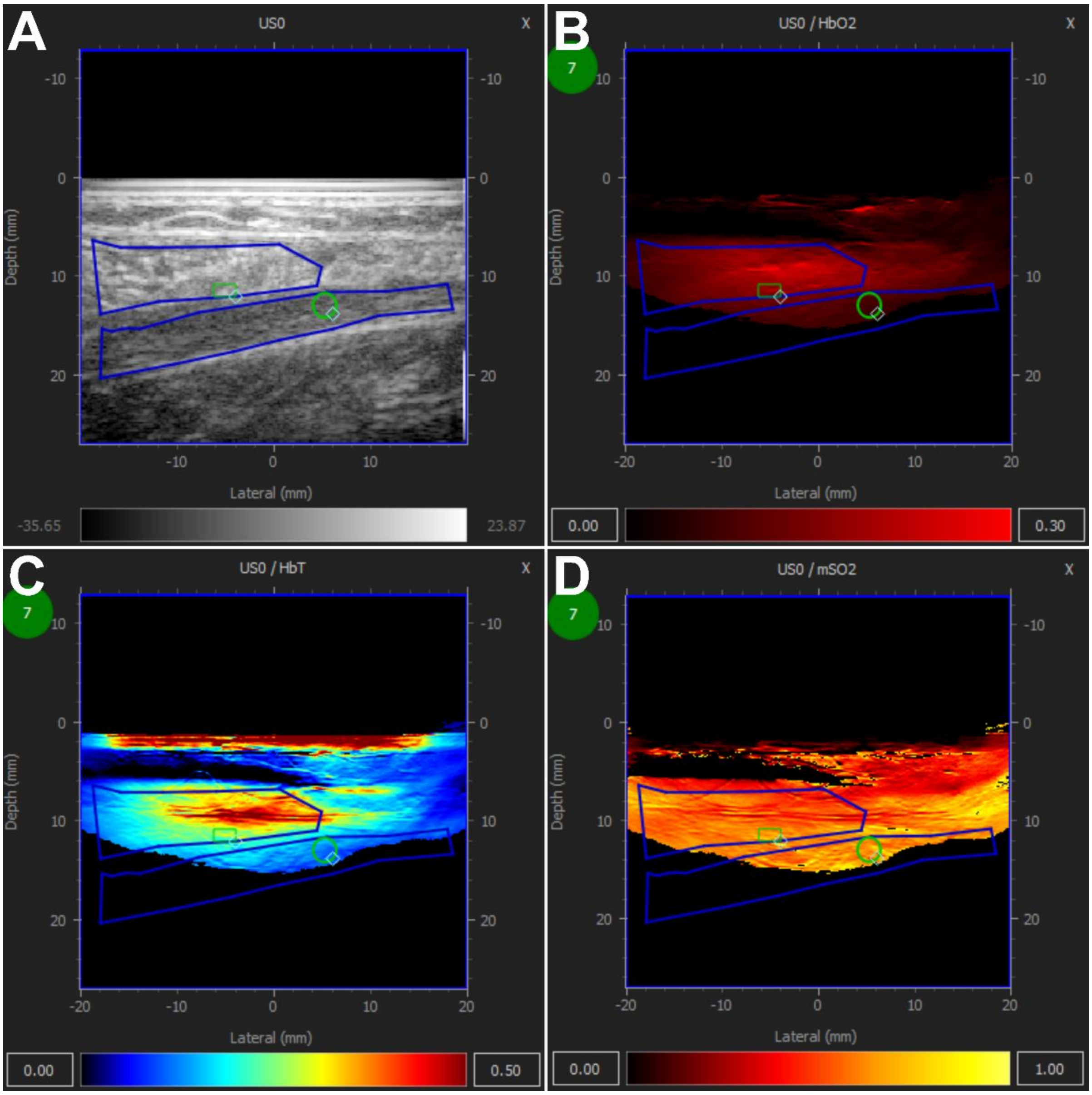

Figure S31. Example of a sagittal MSOT image of the medial gastrocnemius muscle (upper ROI) and aponeurosis (lower ROI) on the injured side of a muscle strain injury patient (MSI). A) B-mode. B) Oxygenated hemoglobin. C) Total hemoglobin. D) Oxygen saturation. ROIs delineating the entire visible tendon (blue) and a smaller central region (green) are shown.

MSI – Injured – Collagen, lipid – Axial

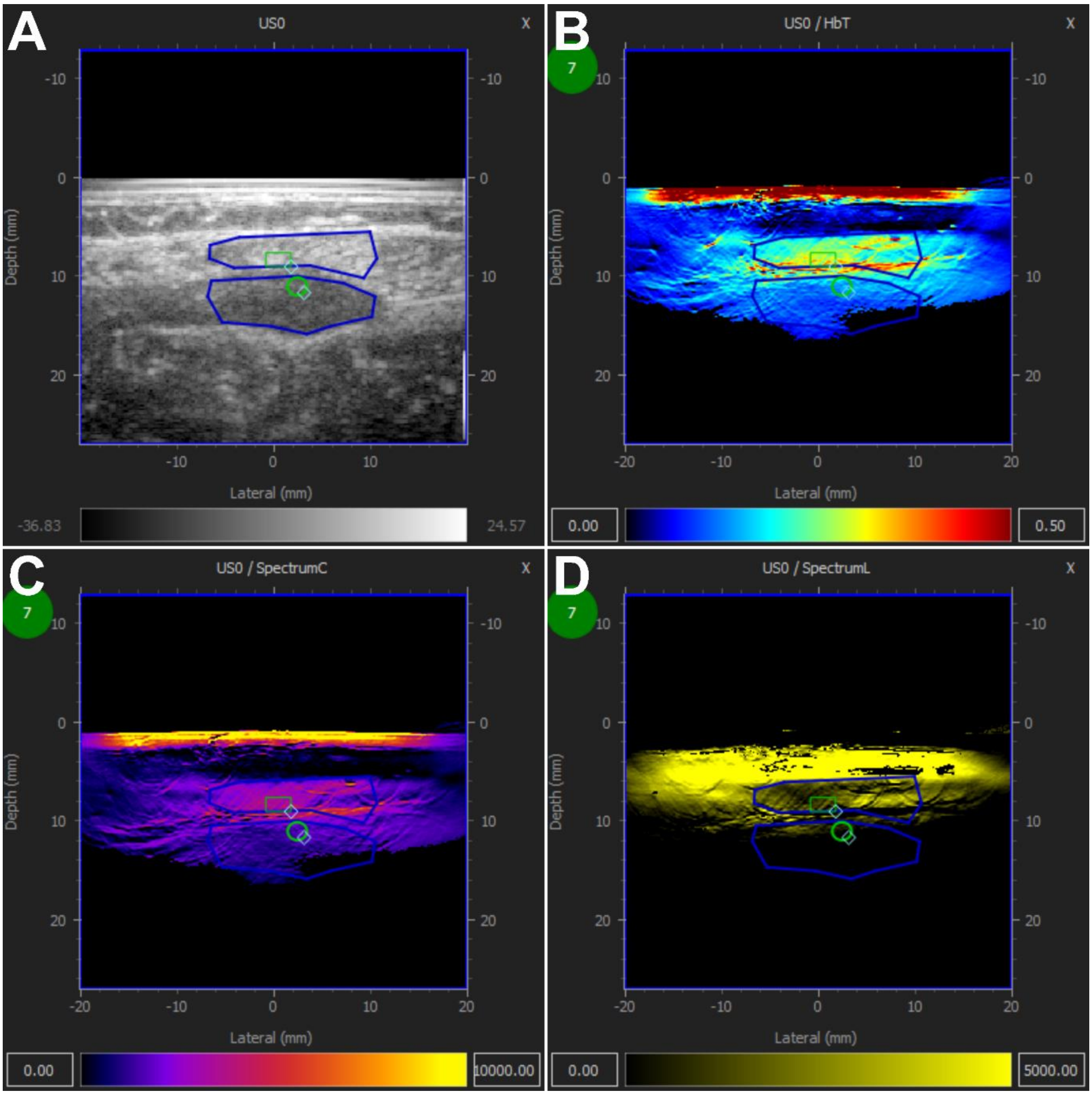

Figure S32. Example of an axial MSOT image of the medial gastrocnemius muscle (upper ROI) and aponeurosis (lower ROI) on the injured side of a muscle strain injury patient (MSI). A) B-mode. B) Total hemoglobin. C) Collagen (Spectrum C). D) Lipid (Spectrum L). ROIs delineating the entire visible tendon (blue) and a smaller central region (green) are shown.

MSI – Injured – Blood – Axial

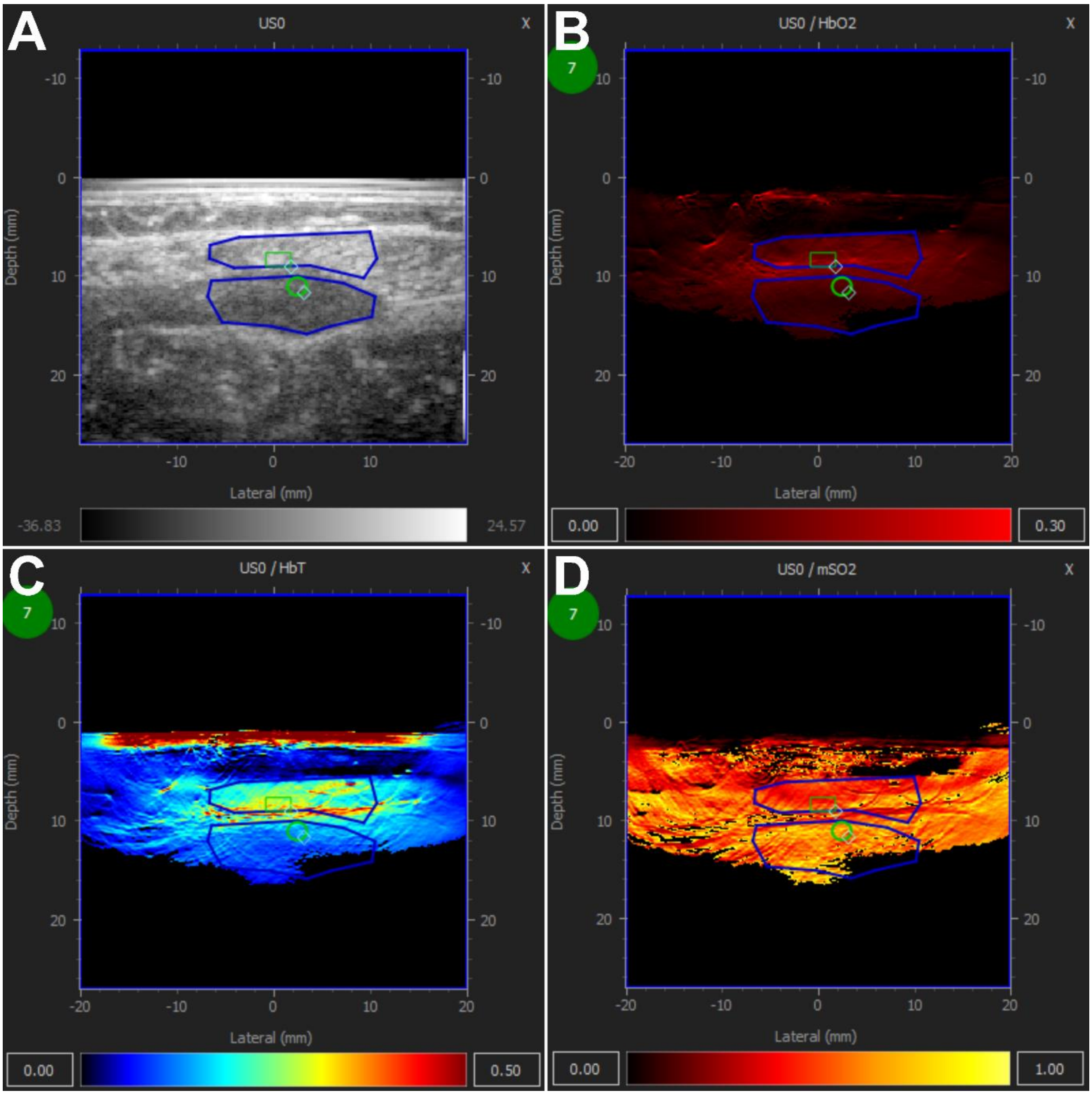

Figure S33. Example of an axial MSOT image of the medial gastrocnemius muscle (upper ROI) and aponeurosis (lower ROI) on the injured side of a muscle strain injury patient (MSI). A) B-mode. B) Oxygenated hemoglobin. C) Total hemoglobin. D) Oxygen saturation. ROIs delineating the entire visible tendon (blue) and a smaller central region (green) are shown.
